# Enhancing co-translational folding of heterologous protein by deleting non-essential ribosomal proteins in *Pichia pastoris*

**DOI:** 10.1186/s13068-019-1377-z

**Published:** 2019-02-21

**Authors:** Xihao Liao, Jing Zhao, Shuli Liang, Jingjie Jin, Cheng Li, Ruiming Xiao, Lu Li, Meijin Guo, Gong Zhang, Ying Lin

**Affiliations:** 1 Guangdong Key Laboratory of Fermentation and Enzyme Engineering, School of Biology and Biological Engineering, South China University of Technology, Guangzhou, 510006 China; 2 Guangdong Research Center of Industrial Enzyme and Green Manufacturing Technology, School of Biology and Biological Engineering, South China University of Technology, Guangzhou, 510006 China; 3 Key Laboratory of Functional Protein Research of Guangdong Higher Education Institutes, Institute of Life and Health Engineering, College of Life Science and Technology, Jinan University, Guangzhou, 510632 China; 4 State Key Laboratory of Bioreactor Engineering, East China University of Science and Technology, Shanghai Institute of Biomanufacturing Technology & Collaborative Innovation Center, Shanghai, 200237 China

**Keywords:** Heterologous protein, *Pichia pastoris*, Ribosomal protein, Translation, Co-translational folding

## Abstract

**Background:**

Translational regulation played an important role in the correct folding of heterologous proteins to form bioactive conformations during biogenesis. Translational pausing coordinates protein translation and co-translational folding. Decelerating translation elongation speed has been shown to improve the soluble protein yield when expressing heterologous proteins in industrial expression hosts. However, rational redesign of translational pausing via synonymous mutations may not be feasible in many cases. Our goal was to develop a general and convenient strategy to improve heterologous protein synthesis in *Pichia pastoris* without mutating the expressed genes.

**Results:**

Here, a large-scale deletion library of ribosomal protein (RP) genes was constructed for heterologous protein expression in *Pichia pastoris*, and 59% (16/27) RP deletants have significantly increased heterologous protein yield. This is due to the delay of 60S subunit assembly by deleting non-essential ribosomal protein genes or 60S subunit processing factors, thus globally decreased the translation elongation speed and improved the co-translational folding, without perturbing the relative transcription level and translation initiation.

**Conclusion:**

Global decrease in the translation elongation speed by RP deletion enhanced co-translational folding efficiency of nascent chains and decreased protein aggregates to improve heterologous protein yield. A potential expression platform for efficient pharmaceutical proteins and industrial enzymes production was provided without synonymous mutation.

**Electronic supplementary material:**

The online version of this article (10.1186/s13068-019-1377-z) contains supplementary material, which is available to authorized users.

## Background

*Pichia pastoris* is a widely used platform for heterologous protein expression, which is a “generally regarded as safe” (GRAS) microorganism. Unlike the bacterial expression systems, which lack the modification enzymes, *Pichia pastoris* is able to produce heterologous proteins with post-translational modifications, especially glycosylation, which is crucial for optimal properties of many pharmaceutical proteins. Over 5000 proteins were manufactured in *P. pastoris* (data from RCT *Pichia* technology), most of which are industrial enzymes and biopharmaceutical proteins [1, 2]. However, due to the remarkable codon preference and tRNA content against the other species, heterologous proteins expressed in *Pichia pastoris* often encounter folding problem, which severely limited their production efficiency [3]. To solve this problem, current strategies mainly focus on codon optimization, protein refolding, and secretion pathway engineering [2, 4, 5]. However, these methods are successful only for a small fraction of proteins, and intensive trials and modifications are often necessary.

Recent studies have revealed that the amino acid sequence does not guarantee correct folding of proteins. The translational pausing sites, which are mediated by clustered but non-consecutive slow-translating codons, coordinate protein biosynthesis and co-translational folding [6]. Translational pausing sites are highly correlated with protein structural domains [7–9]. Therefore, rational design of translational pausing sites via synonymous substitutions may largely enhance the folding efficiency of heterologous proteins and thus yield large amount of active proteins [10, 11]. Although the small proteins with robust folding landscape and rigid native structure do not need translational pausing to fold correctly [12], global perturbation of translational pausing leads to massive aggregation in bacteria [13], demonstrating that a large fraction of the proteins fold in a co-translational folding-dependent manner. For example, green fluorescent protein (GFP) is a eukaryotic protein containing 11 beta-sheet structures and its folding yield was significantly increased by co-translational folding in *E. coli* [14]. Another protein, phytase (Phy) from *Citrobacter amalonaticus* CGMCC 1696, has 95% homology to *E. coli*-derived phytase which consists of one α-domain containing five α-helices and a β-hairpin, and one α/β-domain including seven β-sheets and four α-helices [15]. These proteins are beta-sheet rich or multi-domain and aggregation prone, which may require higher co-translational folding efficiency.

Although a rational design of translational pausing showed its distinguished power in optimizing soluble expression of heterologous proteins, it still necessitates intensive computational and experimental efforts, which often includes numerous synonymous mutations. In addition, rational design requires structural knowledge of the target protein, which is often not available. These obstacles restricted the application of this strategy. Therefore, a more general expression platform is preferred in case that the rational design of translational pausing could not be applied. The microorganisms tend to suppress translation system under stress conditions to save energy, including oxidative stress [16], hypoxia, unfolded protein stress [17–19] and extensive protein expression [20–22]. In *P. pastoris*, overexpression of xylanase A leads to significant down-regulation of numerous ribosomal proteins, including 21 large subunit ribosomal proteins (RPL) and 9 small subunit ribosomal proteins (RPS) [23]. Global deceleration of translation elongation would enhance the folding efficiency of proteins [6, 16] and thus may provide a universal expression platform for heterologous proteins without the necessity of intensive synonymous mutations. Notably, a number of ribosomal proteins (RPs) in *P. pastoris* are non-essential, i.e., deletion strains of these RPs are not lethal, although impairing the growth, indicating that the translation rate is down-regulated. This provided a number of candidates of “slow ribosomes” to enhance the soluble expression of heterologous proteins.

In this study, 27 RP deletion strains of *P. pastoris* were analyzed and two heterologous proteins, eGFP and Phy, driven by the *AOX1* promoter, were expressed in them. RP deletion did not alter the relative mRNA level and translation initiation efficiency. 16 RP deletion strains significantly improved the expression efficiency, indicating that the decelerated translation elongation promoted the co-translational folding of heterologous proteins.

## Results

### Expression efficiency analysis of heterologous protein in RP deletion strains

Due to the incomplete annotation of *P. pastoris* genome, the *P. pastoris* GS115 genome (http://bioinformatics.psb.ugent.be/blast/moderated/?project=orcae_Picpa) was searched for the RP homologs using the RP gene sequences of *S. cerevisiae* [24]. Compared to 79 RPs encoded by 138 genes in *S. cerevisiae* [25], 77 RPs are encoded by 86 genes and the homologous proteins of Rpl27 and Rpl41 from *S. cerevisiae* are missing in *P. pastoris* (Additional file 1: Table S1). Only nine RPs are encoded by a pair of paralogous genes that, in most cases, encode identical or very similar protein products in *P. pastoris* (Additional file 2: Table S2). These findings indicated that the number and composition of the RPs of two budding yeasts are totally different. In this study, 27 RP deletion strains were successfully constructed, which suggest these RPs are non-essential in *P. pastoris* (Additional file 2: Figure S1, Additional file 3: Table S5).

**Fig. 1:**
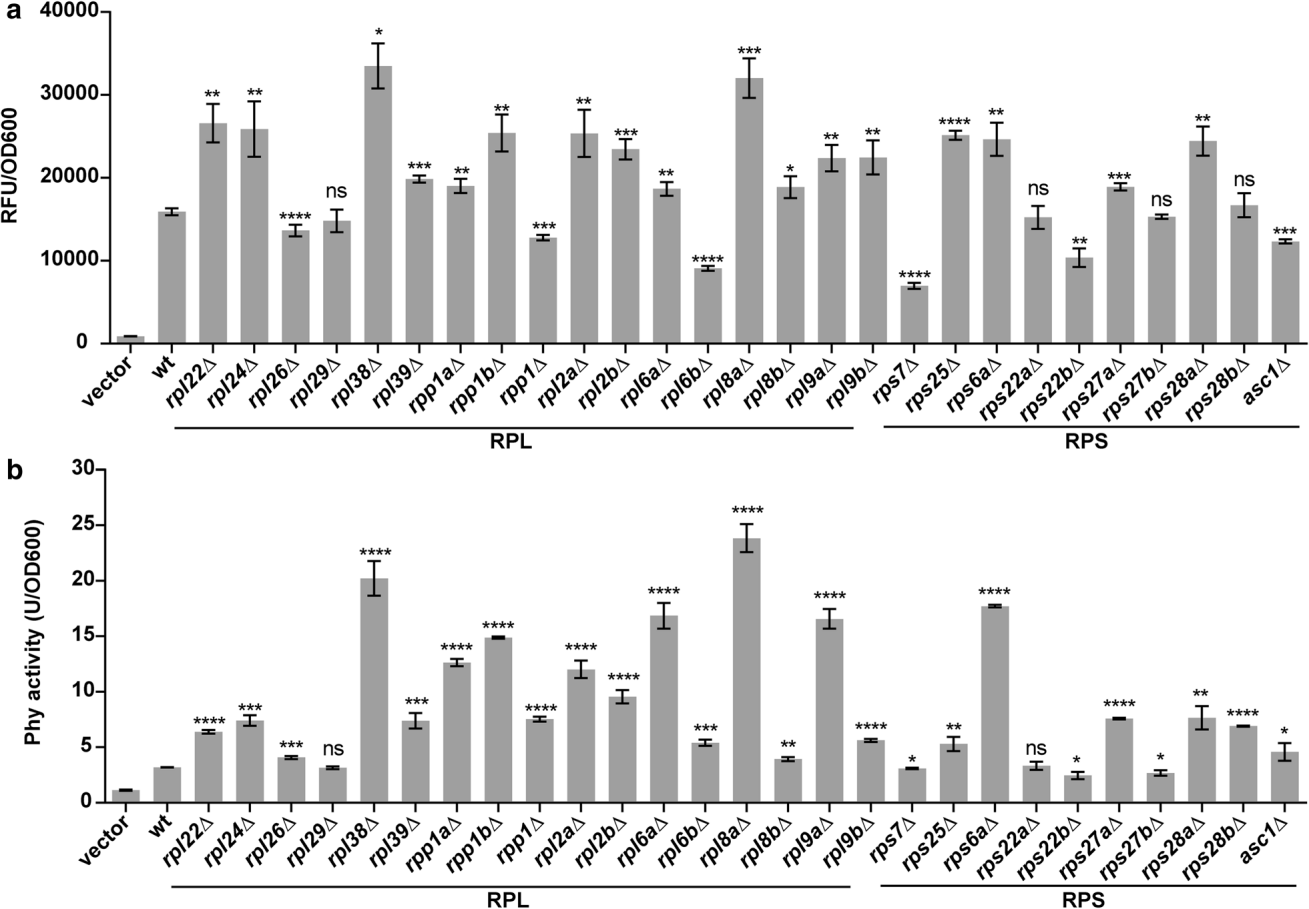
Heterologous protein expression in RP deletion strains. Specific expression of two heterologous proteins (**a** eGFP and **b** Phy) in the wild-type and RP deletion strains. The wild-type strain transformed with empty vector pPICZαA was used as negative control. All strains were fermented in the liquid BMMY for 120 h by feeding 1% methanol per 24 h. Expression levels were measured by the relative fluorescence units (RFU) or enzyme activity and OD_600_. Error bars represent s.d. across three biological replicates. Significance against wild type is indicated as: *p < 0.05; **p < 0.01; ***p < 0.001, *t* test

*P. pastoris* strains were transformed only with low amounts of the *Mss*I-linearized expression cassettes (0.5–1 μg of DNA) to avoid multi-copy integration [26] and yield single copy eGFP or Phy gene in the *P. pastoris* GS115 genome confirmed by the quantitative real-time PCR (Additional file 2: Table S3). In total, 16 RP deletion strains significantly increased the expression of both eGFP and Phy (Fig. 1, Additional file 2: Figure S2). Most of these “enhancing deletion strains” (12 out of 16) were RPL (RP of the large subunit) deletants, indicating that RPLs are important determinants of heterologous protein expression in *P. pastoris*.

### The relative transcription and translation initiation of Phy in RP deletion strains

**Fig. 2:**
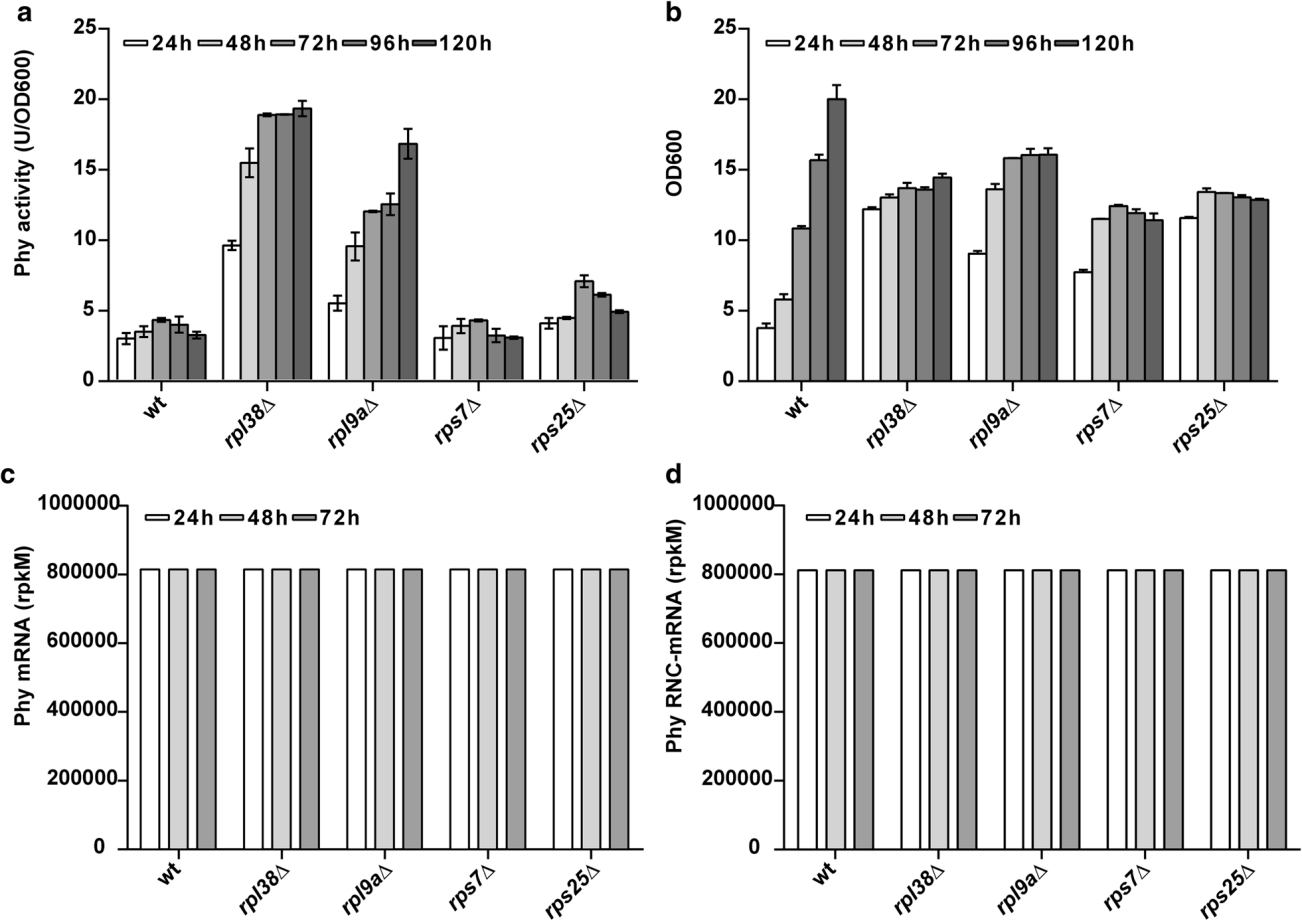
Phy expression in RP deletion strains and wild type. **a**, **b** Phy expression profiles of the *rpl38*∆, *rpl9a*∆, *rps7*∆ and *rps25*∆ strains relative to the wild-type strain. **a** Activity per OD_600_; **b** Growth curve. **c**, **d** Expression of the Phy in transcription level and translation level (RNC-mRNA) of *rpl38*∆, *rpl9a*∆, *rps7*∆, *rps25*∆ and wild-type strains at the early and middle stage of fermentation. Expression levels were measured using next-generation sequencing, evaluated using rpkM as unit

To verify the transcription level and translation initiation efficiency in RP deletion strains, the typical “enhancing” strains of *rpl38*∆, *rpl9a*∆, *rps25*∆ and a “non-enhancing” strain of *rps7*∆ expressing Phy are chosen as representative strains (Fig. 2a, Additional file 2: Figure S3). Also, the growth pattern of RP deletion strains was changed. After 12 h, all strains were already out of exponential phase. The RP deletants grew much slower after 24 h, while the wild-type strain grew constantly till 120 h (Fig. 2b, Additional file 2: Figure S3d). Previous to this, these RP deletion strains were complemented with the corresponding RP actuated by the strongly constitutive promoter *GAP*. The RP complementation strains showed more similar heterologous protein expression relative to the RP deletion strains, indicating that it was actually the RP deletions that caused the observed phenotypes (Fig. 2a, b, Additional file 2: Figures S3, S4). To be noted, the specific activity of Phy in RP-complemented strains was similar to the wild-type level (~ 5 U/OD_600_), much lower than the values (10 ~ 20 U/OD_600_) in *rpl38*∆ and *rpl9a*∆ strains, suggesting that the ribosome performance is the major factor of the specific activity of heterologous protein.

Next, we tested the mRNA abundance and translation initiation of Phy in RP deletion strains. While these strains accumulate heterologous proteins in a time-dependent manner, the relative mRNA level of the Phy remained constant over the time and across the strains, with a standard deviation of only 50 parts per million (ppm), determined by mRNA-seq quantified using reads per kilobase per million reads (rpkM) (Fig. 2c). Meanwhile, the ribosome–nascent chain complex (RNC) mRNA (RNC-mRNA, mRNAs attached to the ribosomes) of Phy, representing the Phy mRNA which entered the translation process, remained also constant over the time and across the strains, with a standard deviation of 153 ppm, determined by RNC-seq (Fig. 2d). These results demonstrated that deletion of the non-essential RPs did not influence the relative transcription and translation engagement of the heterologous mRNA at all.

The above-mentioned results indicated that the “enhancing” RP deletion strains may increase the activity of heterologous proteins by facilitating their folding. To further determine whether the enhanced folding occurs during the translation or after the translation, we analyzed the relative mRNA and RNC-mRNA level of molecular chaperones. In general, RNA-seq and RNC-seq data revealed almost no significant change in the expression level of chaperones (Additional file 4: Tables S6, S7). This implied that the co-translational folding was enhanced.

### Delay of 60S ribosomal subunit assembly enhances heterologous protein expression

**Fig. 3:**
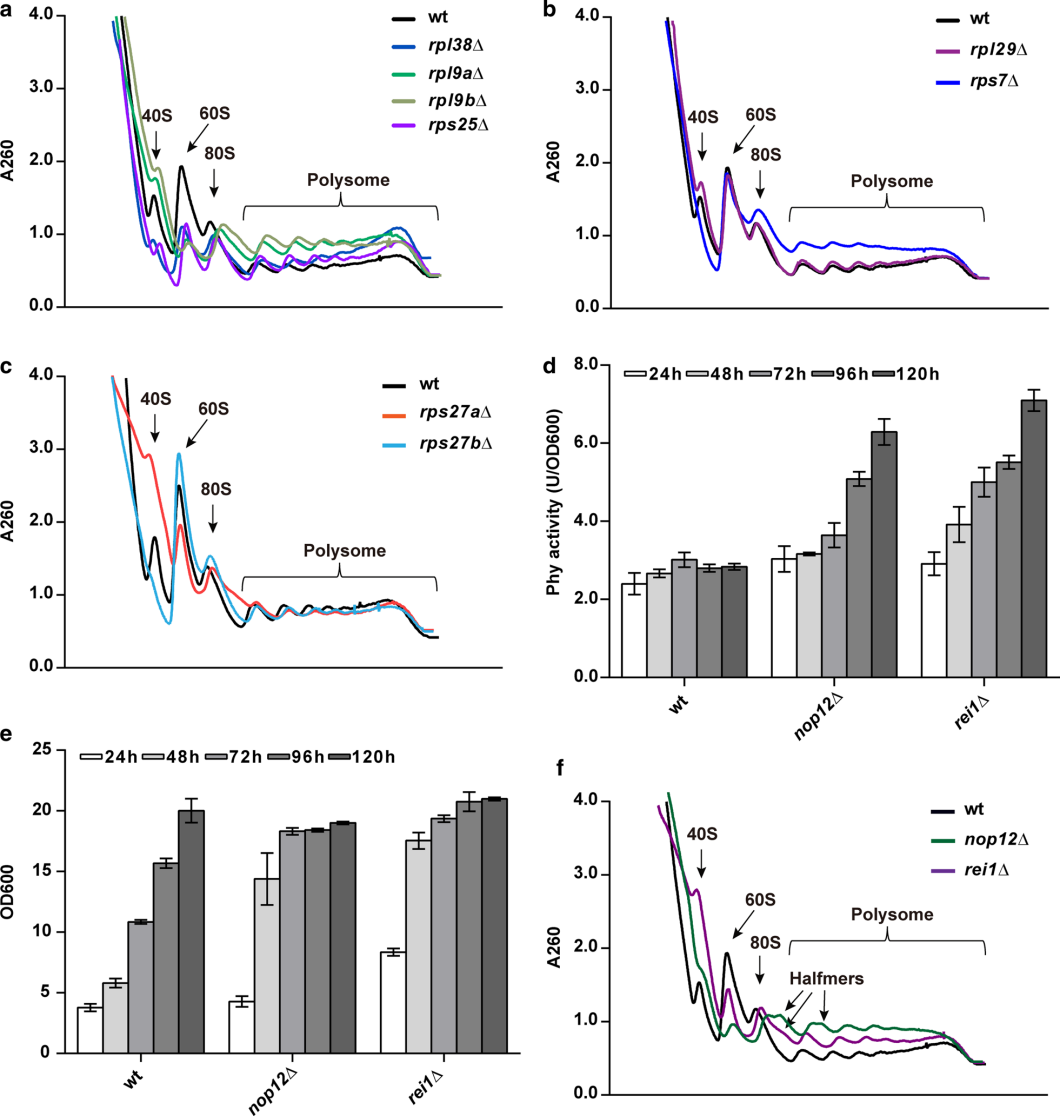
Ribosome assembly in RP deletion strains and 60S processing factor deletion strains, analyzed by polysome profiles. **a**, **b** Polysome profiles of the “enhancing” **a** and “non-enhancing” **b** strains in relation to wild-type strain showed that the former had the lower 60S level. **c** Polysome profiles of RP deletion paralogs. The “enhancing” strain *rps27a*∆ showed a reduced level of 60S ribosomal subunits relative to its paralog deletion strain (“non-enhancing”). **d**, **e** Phy expressional profiles of non-essential 60S processing factor deletion strains relative to wild type. **d** Activity per OD_600_; **e** Growth curve. **f** Relative to wild type, polysome profiles for *rei1*Δ and *nop12*Δ strains showed reduction of the 60S subunit level

Yeast non-essential RPs play important roles in ribosome biogenesis and function [27]. Deletion of particular RPs can delay or impair subunit assembly [27–31]. To evaluate the global impact of RP repression on ribosome assembly and translation, polysome profiles were performed for several RP deletion strains expressing Phy in high-salt conditions (to disrupt non-translating 80S monosomes) (Fig. 3a–c). The “enhancing” strains of *rpl38*∆, *rpl9a*∆, *rpl9b*∆, *rps25*∆ and *rps27a*∆ exhibited lower 60S ribosomal subunits than the wild-type strain (Fig. 3a, c), indicating that the 60S subunit availability is decreased. In contrast, the “non-enhancing” strains, *rpl29*∆, *rps7*∆ and *rps27b*∆, showed almost identical 60S peaks as the wild type (Fig. 3b, c).

To validate this finding, two 60S subunit processing factors were knocked out, *nop12* and *rei1* [32–34], to delay 60S subunit assembly. Both deletion strains showed lower 60S peak in the polysome profiles as expected, and half-mer peaks appeared after monosome/polysome (Fig. 3f), which represent that 48S initiation complexes exist on actively translated mRNAs [31, 35]. Interestingly, the Phy expression in these deletion strains was also enhanced robustly, both measured using unit/OD_600_ (Fig. 3d, e) and unit ml^−1^ (Additional file 2: Figure S5). These results indicated that the 60S assembly delay might be necessary for the expression enhancement of heterologous proteins.

### RP deletion promotes co-translational folding of Phy and decreases protein aggregates

Although the relative number of mRNA molecules involved in the translation did not change (Fig. 2d), decreased availability of 60S subunit would reduce the translation initiation rate, which should result in lower protein production rate. However, the final protein level of Phy was increased. Therefore, the only explanation would be a much lower protein degradation rate, which indicates remarkably better folding efficiency of Phy. According to the “pause to fold” theory [6], this could be only achieved by slower translation elongation speed. Indeed, higher fraction of polyribosomes were observed in the “enhancing” strains, including the *nop12* and *rei1* deletion strains, indicating remarkably slower translation elongation speed along the mRNA [16]. The slower elongation speed could be easily explained by defective 60S subunit.

**Fig. 4:**
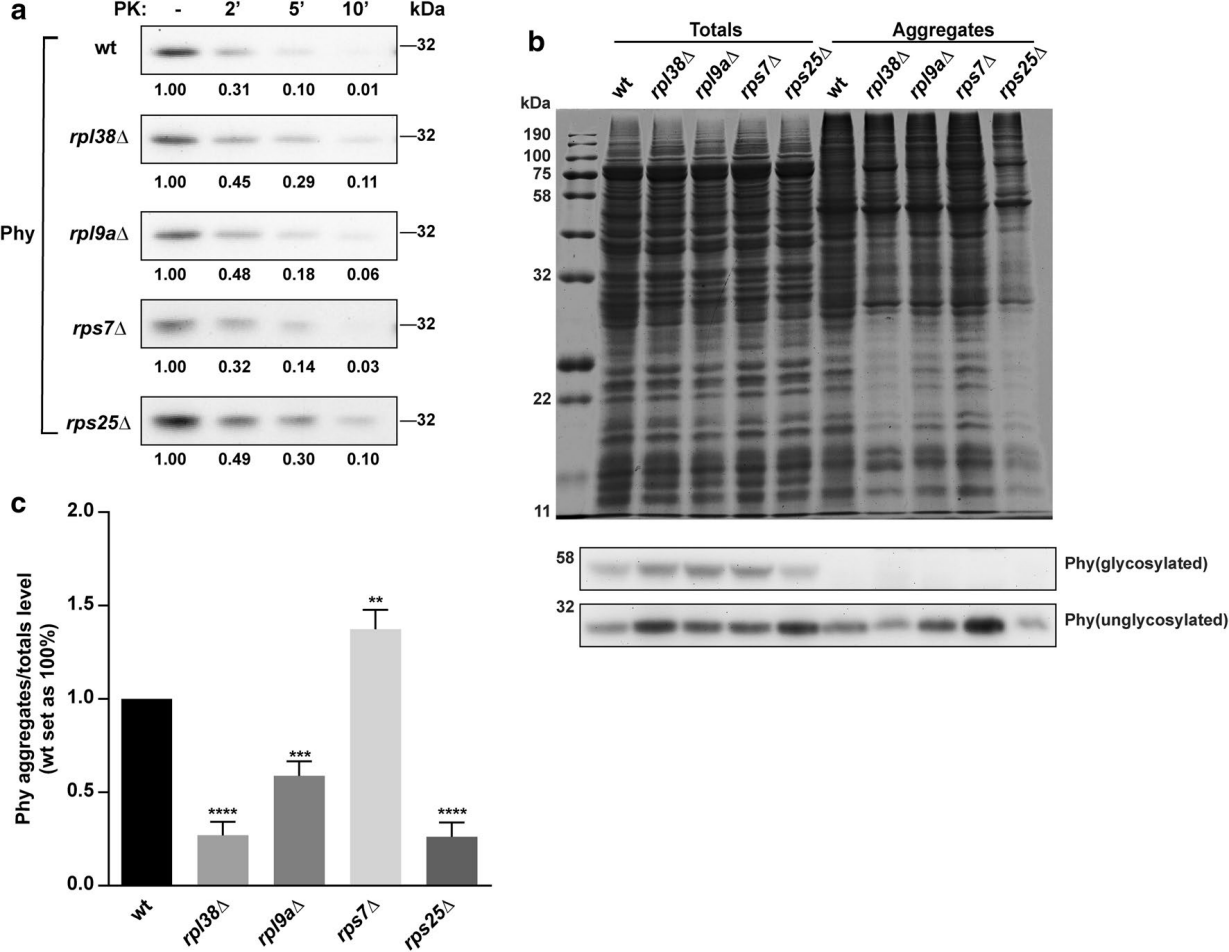
Co-translational folding efficiency of Phy nascent chains and protein aggregates in RP deletion strains. **a** Assessment of co-translational folding efficiency of Phy nascent chains in wild-type and RP deletion strains. After the limited proteinase K digestion, samples were separated by SDS-PAGE, and the remaining Phy was visualized by western blotting. The optical density of Phy band was calculated and the RNC aliquots from the same samples with proteinase K digestion were normalized to those with no proteinase K digestion. **b** Quantitative analysis of protein aggregates extracted from wild-type and RP deletion strains transformed with Phy which is glycosylated in *P. pastoris* [57]. The samples were subject to SDS-PAGE separation followed by Coomassie blue staining, and Phy was visualized by western blotting. **c** Quantification of Phy aggregate level as shown in **b**. The aggregated Phy signals were normalized to totals and wild-type strain transformed with Phy was set as 100%. Unpaired *t* test of GraphPad Prism 6 was used for statistical analysis. Significant difference of Phy aggregates level is indicated as: *p < 0.05; **p < 0.01; ***p < 0.001. Error bars represent s.d. of at least three independent experiments

To further validate the “pause to fold” hypothesis in our case, Phy with the N-terminal HA-tag was expressed to assess the proteolytic susceptibility of Phy nascent chains, which reflects the co-translational folding efficiency, similar to our previous work [10]. After 12 h induction by methanol, the RNCs were isolated and then digested using proteinase K. The Phy nascent chains in “enhancing” strains *rpl38*∆, *rpl9a*∆ and *rps25*∆ were significantly proteinase K resistant relative to wild type, showing better co-translational folding efficiency (Fig. 4a, Additional file 2: Table S4). In contrast, the Phy nascent chain in the *rps7*∆ strain was as vulnerable as in the wild-type strain (Fig. 4a, Additional file 2: Table S4).

In another aspect, better co-translational folding efficiency should be accompanied by less protein aggregates. Indeed, the “enhancing” strains of *rpl38*∆, *rpl9a*∆ and *rps25*∆ showed less Phy aggregates than the wild-type strains, while the “non-enhancing” *rps7*∆ showed stronger protein aggregates of intracellular total proteins and Phy (Fig. 4b, c). To be on the safe side, our method of isolating insoluble proteins was validated using bovine serum albumin fraction V (BSA) as a representative indicator of soluble proteins (Additional file 2: Figure S6) [36].

Taken together, these data revealed that slower translation elongation speed caused by the non-essential RP deletion enhanced heterologous protein expression by increasing the co-translational folding efficiency of nascent chains and decreasing protein aggregates.

## Discussion

In *S. cerevisiae*, 60S subunit assembly defect by RP deletion increases Gcn4 expression to repress translation and extend life span [29, 31]. In this study, defective 60S subunit assembly by RP deletion reduced protein aggregates of both heterologous (Fig. 4c) and endogenous (Fig. 4c) proteins. Although aggregation of endogenous proteins could be a result of expressing the heterologous protein, these findings imply that lower protein aggregates by RP deletion may be beneficial to save energy and increase yeast life span.

In the excellent hosts for recombinant protein production, such as *E. coli* and *P. pastoris*, one of the bottlenecks for most heterologous protein synthesis is protein folding [37, 38]. In protein synthesis, folding quality control in the stage of co-translation and post-translation is crucial for forming the native conformation and decreasing protein misfolding and aggregation [39–41]. Current strategies for improving heterologous protein folding mainly focused on the past-translational phase, including co-expression of chaperones and helpers in cytoplasm or endoplasmic reticulum and glycosylation engineering in *P. pastoris* [3–5, 42–44]. However, a proteome-wide study of chaperonin-dependent protein folding indicates that folding of many proteins is not strictly dependent on molecular chaperones like GroEL/ES [45]. In this paper, the mRNA-seq and RNC-seq data showed that the abundance of genes encoding chaperones was not significantly increased to the level observed in the “enhancing” strains, suggesting that the post-translational folding executed by chaperones may not change (Additional file 4: Tables S6, S7). Besides, approximately 70% of newly translated polypeptides rely on co-translational folding of ribosome-associated Hsp70 in eukaryotes, which are, mostly, long, multi-domain, beta-sheet-rich, aggregation-prone, slow-translational, and slower folded proteins [41]. The reporter proteins eGFP and Phy used in this study meet the feature of slower folded proteins [14, 15], which may require slow translation elongation rate for co-translational folding. Thus, an overall decrease in elongation speed by deletion of non-essential RP improved their co-translational folding efficiency (Fig. 4a). More importantly, these deletion strains create an optimized translation scenario for heterologous proteins. This is convenient for heterologous protein production without a priori knowledge of protein structures and rational design of translational pausing sites [10, 12].

Intriguingly, the higher fraction of polyribosomes did not guarantee a slower elongation speed. The *rps7*∆ strain exhibited a higher fraction of polyribosomes (Fig. 3b). However, the Phy expression was not enhanced and more protein aggregates were found in the *rps7*∆ strain, indicating that the translation elongation was not decelerated. A reasonable explanation would be that the *rps7*∆ strain possesses more ribosomes. Indeed, more rRNAs were found in the *rps7*∆ strain (Additional file 2: Figure S7). More ribosomes resulted in higher translation initiation rate. Considering that the relative mRNA level is not changed (Fig. 2c), higher translation initiation rate will lead to more ribosomes in the polysome fraction [46]. This is echoed by the fact that the *rps7*∆ strain showed the same 60S subunit peak as the wt, while all “enhancing” strains showed lower 60S ribosomal subunit than wt (Fig. 3).

In addition, codon optimization, which replaces rare codons with the preferred codons without amino acid alteration, can accelerate translation and may enhance heterologous protein production [47]. For example, the enzyme activity of a codon-optimized endoglucanase gene from *Trichoderma reesei* was increased 24% relative to the natural gene in *P. pastoris* [48]. However, codon optimization perturbs the co-translational folding of many proteins because of translation initiation or elongation acceleration, which may cause protein misfolding and limit the production of heterologous protein [49, 50]. In this work, enhancing the co-translational folding of heterologous protein by RP deletion greatly improved the expression of codon-optimized eGFP and Phy in *P. pastoris*, suggesting that these RP deletion strains were suitable for decreasing the translation rate of the codon-optimized gene, thus increasing the yield of soluble and bioactive proteins while lowering the cost of manufacturing. Furthermore, many heterologous genes have been transformed in *Pichia pastoris* for biofuel production in recent years [51–53]. A convenient platform was developed to improve heterologous protein expression in *Pichia pastoris*, which may contribute to heterologous pathway expression for synthetic biology applications, such as biofuel production.

## Conclusions

In conclusion, the “slow ribosomes” were constructed to enhance the soluble expression of heterologous proteins based on improved co-translational folding in *P. pastoris*. Loss of non-essential ribosomal proteins increased heterologous protein synthesis without changing the relative transcription level and translation ratio of heterologous protein. Further studies found that 60S subunit assembly defect was a key determinant of increased heterologous protein yield with the improved co-translational folding efficiency of nascent chains. According to the principle of “pause to fold” which is slowing down the elongation speed at a certain region of mRNA coordinates co-translation folding and protein synthesis [6, 46, 54–56], defective 60S subunit assembly by RP deletion enhances co-translational folding efficiency of nascent chains and decreases protein aggregates to improve heterologous protein yield. Therefore, our work opens an avenue for improving heterologous protein production based on elaborate protein quality control. Further studies will focus on the validation of these RP deletion strains in more pharmaceutical proteins and industrial enzymes.

## Methods

### Strains and plasmids

*Escherichia coli* strain TOP10 was used for recombinant DNA manipulation. *Pichia pastoris* GS115 (Invitrogen) expressing eGFP or Phy was used as the wild-type (wt) strain and all deletion strains used in this study were derived from it. The pPICZαA (Invitrogen) vector including *AOX1* promoter was used to express the heterologous protein of eGFP and Phy from *Citrobacter amalonaticus* CGMCC 1696 [57]. The pGAPZA (Invitrogen) vector containing *GAP* promoter was used to construct the RP complementation strains.

### Transformation and cultivation conditions

Electroporation was used for *P. pastoris* transformation and transformants were screened on YPDSZ (1% yeast extract, 2% peptone, 2% glucose, 2% agar, 1 M sorbitol, 100 µg ml^−1^ Zeocin). To avoid multi-copy integration, the method from Thomas et al. was employed [26]. The plasmids containing eGFP or Phy gene were linearized by *Mss*I enzyme. Then low amounts of *Mss*I-linearized expression cassettes (0.5 to 1 μg of DNA) were transformed into *P. pastoris* strains. After verification by PCR, positive transformants were cultivated in a liquid medium BMGY [1% yeast extract, 2% peptone, 1.34% YNB (yeast nitrogen base without amino acids), 1% glycerol, 100 mM potassium phosphate (pH 6.0)] for 20–24 h (2 to 6 OD_600_) in a 50 ml shake flask. Then the strains were washed with 100 mM potassium phosphate (pH 6.0) or sterile water and transferred to 250 ml shake flask containing 25 ml BMMY [1% yeast extract, 2% peptone, 1.34% YNB, 1% methanol, 100 mM potassium phosphate (pH 6.0)] with initial OD_600_ = 1.0. The fed-batch fermentation was proceeded to express eGFP and Phy for 120 h by feeding 1% methanol per 24 h. All liquid culture was performed at 30 °C and 250 rpm. The samples for RNA-seq and RNC-seq were taken before adding methanol at 24, 48 and 72 h after growth in BMMY medium with an initial OD_600_ of 1.0. In addition, the samples for polysome profiles, determination of nascent chain folding, and isolation of protein aggregates were taken at 12 h in the same cultivation conditions.

### Construction of RP and 60S assembly factor deletion strains

Deletants were generated by PCR-mediated gene disruption based on homology recombination as follows [58]. The Cre/mutated *lox* system was used for recycling the Zeocin (Invitrogen) resistance marker. The Cre-ZeoR cassette including *cre* gene amplified from the plasmid pSH47 and *Sh ble* gene was constructed by ligating the *EcoR*I/*Sac*II-digested fragment of *cre* gene to pPICZA (Invitrogen). Then overlapped 30–40 bp nucleotides were designed to fuse the Cre-ZeoR cassette with two homology fragments, which is the flank of the deleted region. The purified fusion PCR products were transformed into the competent cells of *P. pastoris* by electroporation with the parameters set at 1.5 kV, 200 ω and 25 µF. YPDSZ plates were used to screen for Zeocin-resistant transformants and positive transformants were identified by PCR. To recycle the Zeocin resistance marker, positive transformants were shifted to YPM liquid medium (1% yeast extract, 2% peptone, 1% methanol) for induction culture up to 96–120 h by feeding 1% methanol per 24 h. Then the cells were streaked after methanol induction on the YPD plates. Single colonies were diluted in sterile water and then spotted on YPD and YPDZ (YPD plus 100 µg ml^−1^ Zeocin) plates to test whether the Cre-ZeoR cassette had been removed. Finally, the marker-removed deletants were confirmed by PCR. All primers were listed in Additional file 3: Table S5.

### Isolation of ribosome–nascent chain complexes

The method of ribosome–nascent chain complexes (RNCs) extraction was generated as described before with appropriate revision [59, 60]. *P. pastoris* strains (10–20 OD_600_) were pre-treated with 100 µg ml^−1^ cycloheximide (Sigma-Aldrich) for 5 min at 30 °C and 250 rpm and washed with 20 mM potassium phosphate (pH 7.4), followed by incubation with 1 ml buffer I (20 mM potassium phosphate (pH 7.4), 10 mM DTT (or 0.5% β-mercaptoethanol), and 100 µg ml^−1^ cycloheximide) for 10 min at 30 °C. After collecting cells by centrifugation (3000*g*) for 3 min at 4 °C, pellets were resuspended with 1 ml buffer II (20 mM potassium phosphate (pH 7.4), 1.2 M sorbitol, 0.5 mM MgCl_2_, 150 U ml^−1^ lyticase (Yeasen, shanghai, China), 100 µg ml^−1^ cycloheximide) and incubated on ice for 10 min. Each sample was divided into two aliquots to extract RNC-RNA and total RNA. For RNCs extraction, once more pellets were collected by centrifugation (3000*g*) for 3 min at 4 °C, followed by adding 1 ml ribosome buffer (10 mM Tris–HCl (pH 7.4), 5 mM MgCl_2_, 100 mM KCl, 2 mM DTT, 100 µg ml^−1^ cycloheximide) with 1% Triton X-100. After 10 min ice bath, cell lysates were centrifuged at 20,000*g* for 10 min at 4 °C to remove cell debris. Supernatants were transferred on the surface of 12.5 ml sucrose buffer (30% sucrose in ribosome buffer). RNCs were pelleted by ultracentrifugation (185,000*g*) in a Type 70 Ti rotor (Beckman Coulter) for 5 h at 4 °C. Another aliquot was suspended with 1 ml TRIzol Reagent (Invitrogen) for total RNA extraction.

### RNA extraction, RNA sequencing and data analysis

The total RNA and RNC-RNA extraction was carried out as previously described [60]. In brief, isolation of total RNA and RNC-RNA was used by TRIzol Reagent, according to the manufacturer’s instructions. Two biological replicates of total RNA and RNC-RNA were performed for subsequent RNA-seq, respectively. The polyA + mRNA was selected from the total RNA and RNC-RNA samples by RNA Purification Beads (Illumina). The cDNA library products were generated using NEBNext Ultra II RNA library prep kit (NEB) and sequenced using the Illumina HiSeq X Ten. Library construction and sequencing were performed at Shenzhen Chi-Biotech Corporation. High-quality reads were kept for the sequence analysis by the Illumina quality filters. The raw sequencing data are available at Gene Expression Omnibus database (accession number GSE116415). The mRNA and RNC-mRNA abundance was normalized using rpkM [61]. Genes with > 10 mapped reads were considered as quantified genes [62]. The edgeR package method was adopted to analyze the differential expression genes [63].

### Polysome profiles

The polysome profiles were performed by modification of the previously described methods [29, 64]. *P. pastoris* strains were cultured in liquid medium BMMY for 12 h and pre-treated with 100 µg ml^−1^ cycloheximide for 15 min at 30 °C, 250 rpm. Then cells were collected by centrifugation (3000*g*) for 3 min at 4 °C, washed with 5 ml lysis buffer (20 mM Tris–HCl (pH 7.4), 5 mM MgCl_2_, 140 mM KCl, 1 mM DTT, 1% Triton X-100, 100 µg ml^−1^ cycloheximide) and suspended in 1 ml lysis buffer. Cells were transferred and lysed by MiniBeadbeater (Biospec products) with 730 mg acid-washed glass beads (Sigma-Aldrich) for 6 × 30 s times at intervals of 1 min on ice. The step of lysis was executed in a 4 °C cold room. Lysates were clarified by centrifugation (3000*g*) for 5 min at 4 °C, and transferred to 1.5 ml tubes for further centrifugation (10,000*g*) for 10 min. 20 (or 25, Fig. 3c) A260 units were loaded on the surface of 10–50% linear sucrose gradients (50 mM Tris–HCl (pH 7.4), 15 mM MgCl_2_, 800 mM KCl, 100 µg ml^−1^ cycloheximide) and ultracentrifuged at 39,000 rpm at 4 °C by SW40 Ti rotor (Beckman Coulter) for 2 h. Finally, gradients were collected from the top and measured at 260 nm by Gradient Station (Biocomp).

### Determination of nascent chain folding state

Firstly, cells were grown in BMMY with an initial OD_600_ = 1.0 for 12 h. RNCs pellet was isolated and softly resuspended in 100 µl of the ribosome buffer (10 mM Tris–HCl (pH 7.4), 5 mM MgCl_2_ and 100 mM KCl). All samples were monitored at 260 nm immediately and adjusted to 10 A260 units ml^−1^. 20 µl sample was digested by gently mixing with 0.8 ng µl^−1^ proteinase K and incubated on ice for 2, 5 and 10 min, respectively. Then the reactions were mixed immediately with SDS loading buffer and heated at 100 °C for 5 min. The fraction of nascent chain was analyzed by 15% SDS-PAGE and visualized by western blot. The ImageJ software was used to quantify the bands.

### Isolation of protein aggregates

The procedure of protein aggregates was prepared according to a previously reported protocol [65]. 50 OD_600_ units of 12 h-inductional strains in BMMY were harvested by centrifugation (3000*g*) for 3 min at 4 °C, and pellets were flash frozen in liquid nitrogen. Pellets were washed with 20 mM potassium phosphate (pH 6.8) and resuspended in buffer II [20 mM potassium phosphate pH 6.8, 1 mM EDTA, 10 mM DTT, 0.1% Tween 20, protease inhibitor cocktail (MedChemExpress), 1 mM PMSF (Yeasen, shanghai, China), 150 U ml^−1^ lyticase and 1.25 U ml^−1^ Benzonase (Yeasen, shanghai, China)], incubated for 15 min at 30 °C and chilled on ice for 5 min. After sonication, the samples were collected by centrifugation (200*g*) for 20 min. The supernatants were diluted to identical protein concentrations and taken as input control (total). Aggregates were sedimented by centrifugation (16,000*g*) for 20 min at 4 °C. The aggregated proteins were washed twice with buffer II (20 mM potassium phosphate pH 6.8, 2% (v/v) NP-40, 1 mM PMSF, protease inhibitor cocktail), sonicated and centrifuged at 16,000*g* at 4 °C for 20 min. Protein aggregates were sonicated with rehydration buffer (7 M urea, 2% CHAPS, 2 M thiourea, 20 mM DTT, 1% SDS) [36], boiled in SDS sample buffer, together with totals separated by 12% SDS-PAGE, and analyzed by Coomassie blue staining and western blotting.

## Additional files

**Additional file 1: Table S1.** Predicted ribosomal protein genes of *P. pastoris* GS115.

**Additional file 2: Table S2.** Ribosomal protein gene paralogs in *P. pastoris* GS115. **Table S3.** Gene copy numbers of eGFP and Phy in *P. pastoris* GS115 genome detected by real-time qPCR. **Table S4.** Two-way ANOVA on three biological replicates of the assay of Phy nascent chains co-translational folding efficiency. **Figure S1.** Confirmation of the deletants by PCR. After methanol induction, single colonies were isolated by streak plate method. Deletants were subject to PCR analysis using two outer primers (e.g., REI1-KO-S and REI1-KO-A), both of which were located outside of the homologous region. An expected deleted fragment could be amplified from deletants using the wide type strains as control. The gene size of RPGs and 60 processing factors is as follows: *rpl22* (390 bp), *rpl24* (477 bp), *rpl26* (384 bp), *rpl29* (180 bp), *rpl38* (237 bp), *rpl39* (156 bp), *rpp1a* (321 bp), *rpp1b* (324 bp), *rpp1* (321/324 bp), *rpl2a* (765 bp), *rpl2b* (825 bp), *rpl6a* (543 bp), *rpl6b* (504 bp), *rpl8a* (753 bp), *rpl8b* (872 bp), *rpl9a* (576 bp), *rpl9b* (576 bp), *rps7* (567 bp), *rps25* (327 bp), *rps6a* (717 bp), *rps22a* (393 bp), *rps22b* (686 bp), *rps27a* (579 bp), *rps27b* (249 bp), *rps28a* (204 bp), *rps28b* (204 bp), *asc1* (1286 bp), *nop12* (1338 bp), *rei1* (1224 bp). **Figure S2.** SDS-PAGE analysis of Phy expression with the collected culture supernatant in RPG deletion strains relative to wild-type at 120 h induction time. The molecular weight (MW) of glycosylated Phy is approximately 55 kDa. **Figure S3.** Expression analysis of two heterologous proteins in four RP deletion strains and wild-type. **a** SDS-PAGE analysis of Phy expression with the collected culture supernatant in *rpl38*∆, *rpl9a*∆, *rps7*∆, *rps25*∆ strains relative to wild-type at 120 h induction time. **b** Phy expression profiles (activity per ml culture) of *rpl38*∆, *rpl9a*∆, *rps7*∆, *rps25*∆ strains relative to the wild-type strain. **c**, **d** eGFP expression profiles and growth curve of *rpl38*∆, *rpl9a*∆, *rps7*∆, *rps25*∆ strains in relation to wild-type strain. **Figure S4.** RP complementation assay with eGFP and Phy expression profiles in RP deletion strains relative to wild-type. **a**, **b** eGFP expression profiles and growth curve. **c**, **d** Phy expression profiles and growth curve. The *RPL38*, *RPL9A*, *RPS7*, *RPS25* gene were amplified by PCR using the primers shown as (Additional file 3: Table S5) from *P. pastoris* GS115 genome. Seamless Assembly Cloning Kit (C5891-25, CloneSmarter, USA) was used to clone these genes into the pGAPZA plasmid. The GAP-RP cassettes were amplified by the specific primers to assemble into the pPICZαA-eGFP and pPICZαA-Phy plasmid, respectively. Then RP deletion strains were transformed with the *Mss*I-linearized expression cassettes to yield RP-complemented strains. **Figure S5.** Phy expression profiles of the 60S processing factor deletion strains relative to the wild-type strain. **a** SDS-PAGE analysis of the collected culture supernatant at 120 h induction time. **b** Activity per ml culture. **Figure S6.** Validation of the method used in isolation of protein aggregates. **a** BSA removal assay. Aggregated protein extraction was performed with total protein and total protein mixed with 10% BSA, separately. SDS-PAGE assay was carried out to visualize the protein band. The first lane was loaded with pure BSA as positive control and the other lane with the dashed boxes indicated the bands of approximate MW of BSA. **b** Quantitative analysis of BSA removal efficiency. The BSA optical density ratios were revealed through normalizing the optical density values of the bands with known BSA additions to those without BSA additions. **Figure S7.** Semi-quantitative analysis of ribosomal RNA in *rps7*∆ strain and wild-type. After methanol induction for 12 h, total RNA was extracted with the same OD_600_ of *rps7*∆ strain and wt. The concentrations of total RNA of the replicate 1: 749 ng µl^−1^ (wt), 1049 ng µl^−1^ (*rps7*∆), and the replicate 2: 738 ng µl^−1^ (wt), 1209 ng µl^−1^ (*rps7*∆) were measured using a Nano-drop spectrophotometer. Finally, the same volume of the sample was analyzed by 2.5% agarose gel electrophoresis.

**Additional file 3: Table S5.** Primers used in this study.

**Additional file 4: Table S6.** mRNA fold-change (log2) for chaperone genes. **Table S7.** RNC-mRNA fold-change (log2) for chaperone genes. P-values of differential expression were calculated using edgeR software.

## Abbreviations

RP: ribosomal protein
RPL: large subunit ribosomal protein
RPS: small subunit ribosomal protein
eGFP: enhanced green fluorescent protein
Phy: phytase
RNC: ribosome–nascent chain complex
RFU: relative fluorescence unit
rpkM: reads per kilobase per million reads
ppm: parts per million
