# Supplementary material for: Enhancing co-translational folding of heterologous protein by deleting non-essential ribosomal proteins in Pichia pastoris
Source: Biotechnol Biofuels. 2019 Feb 21;12:38. doi: 10.1186/s13068-019-1377-z (PMC6383220; doi:10.1186/s13068-019-1377-z)
Supplement: Supplementary file 2 — Additional file 2: Table S2. Ribosomal protein gene paralogs in P. pastoris GS115. Table S3. Gene copy numbers of eGFP and Phy in P. pastoris GS115 genome detected by real-time qPCR. Table S4. Two-way ANOVA on three biological replicates of the assay of Phy nascent chains co-translational folding efficiency. Figure S1. Confirmation of the deletants by PCR. After methanol induction, single colonies were isolated by streak plate method. Deletants were subject to PCR analysis using two outer primers (e.g., REI1-KO-S and REI1-KO-A), both of which were located outside of the homologous region. An expected deleted fragment could be amplified from deletants using the wide type strains as control. The gene size of RPGs and 60 processing factors is as follows: rpl22 (390 bp), rpl24 (477 bp), rpl26 (384 bp), rpl29 (180 bp), rpl38 (237 bp), rpl39 (156 bp), rpp1a (321 bp), rpp1b (324 bp), rpp1 (321/324 bp), rpl2a (765 bp), rpl2b (825 bp), rpl6a (543 bp), rpl6b (504 bp), rpl8a (753 bp), rpl8b (872 bp), rpl9a (576 bp), rpl9b (576 bp), rps7 (567 bp), rps25 (327 bp), rps6a (717 bp), rps22a (393 bp), rps22b (686 bp), rps27a (579 bp), rps27b (249 bp), rps28a (204 bp), rps28b (204 bp), asc1 (1286 bp), nop12 (1338 bp), rei1 (1224 bp). Figure S2. SDS-PAGE analysis of Phy expression with the collected culture supernatant in RPG deletion strains relative to wild-type at 120 h induction time. The molecular weight (MW) of glycosylated Phy is approximately 55 kDa. Figure S3. Expression analysis of two heterologous proteins in four RP deletion strains and wild-type. a SDS-PAGE analysis of Phy expression with the collected culture supernatant in rpl38∆, rpl9a∆, rps7∆, rps25∆ strains relative to wild-type at 120 h induction time. b Phy expression profiles (activity per ml culture) of rpl38∆, rpl9a∆, rps7∆, rps25∆ strains relative to the wild-type strain. c, d eGFP expression profiles and growth curve of rpl38∆, rpl9a∆, rps7∆, rps25∆ strains in relation to wild-type strain. Figure S4. RP complem [file 13068_2019_1377_MOESM2_ESM.docx]

**Additional file 2**

**Table S2. Ribosomal protein gene paralogs in *P. pastoris* GS115.**

| Protein | Gene1 | Gene 2 | Gene length | TOTAL AA length | Number differing AAs |
| --- | --- | --- | --- | --- | --- |
| Rpl2 | *RPL2A* | *RPL2B* | 765/825 | 254 | 0 |
| Rpl6 | *RPL6A* | *RPL6B* | 543/504 | 158/167 | 59 |
| Rpl8 | *RPL8A* | *RPL8B* | 753/872 | 250/243 | 28 |
| Rpl9 | *RPL9A* | *RPL9B* | 576/576 | 191 | 4 |
| Rpp1 | *RPP1A* | *RPP1B* | 321/324 | 106/107 | 47 |
| Rps6 | *RPS6A* | *RPS6B* | 717/801 | 238/236 | 12 |
| Rps22 | *RPS22A* | *RPS22B* | 393/686 | 130 | 5 |
| Rps27 | *RPS27A* | *RPS27B* | 579/249 | 82 | 4 |
| Rps28 | *RPS28A* | *RPS28B* | 204/204 | 67 | 0 |

**Table S3. Gene copy numbers of eGFP and Phy in *P. pastoris* GS115 genome detected by real-time qPCR.**

| Strains | *EGFP* | | *PHY* | |
| --- | --- | --- | --- | --- |
|  | (2^-ΔΔCt^) | Copy number | (2^-ΔΔCt^) | Copy number |
| wt | 1.00±0.11 | 1 | 0.70±0.04 | 1 |
| *rpl38*∆ | 0.36±0.04 | 1 | 1.06±0.12 | 1 |
| *rpl9a*∆ | 0.50±0.13 | 1 | 1.28±0.03 | 1 |
| *rps7*∆ | 0.87±0.07 | 1 | 0.68±0.11 | 1 |
| *rps25*∆ | 0.81±0.20 | 1 | 0.69±0.07 | 1 |

**Table S4. Two-way ANOVA on three biological replicates of the assay of Phy nascent chains co-translational folding efficiency.**

| Samples | Time（mean） | | | | *P* value  （two-way ANOVA） | *P* value summary |
| --- | --- | --- | --- | --- | --- | --- |
|  | - | 2' | 5' | 10' |  |  |
| wt | 1.00 | 0.28 | 0.11 | 0.03 | -- | -- |
| *rpl38*∆ | 1.00 | 0.36 | 0.18 | 0.07 | 0.0387 | * |
| *rpl9a*∆ | 1.00 | 0.40 | 0.18 | 0.11 | 0.0023 | ** |
| *rps7*∆ | 1.00 | 0.31 | 0.13 | 0.02 | 0.3109 | ns |
| *rps25*∆ | 1.00 | 0.49 | 0.21 | 0.07 | <0.0001 | **** |

The ImageJ software was used to quantify the gray scale optical density of western-blot. The mean values were calculated by at least three independent experiments. Two-way ANOVA of Graph Pad Prism 6 was used for statistical analysis. Significant difference of Phy co-translational folding efficiency of RPG deletion strains relative to wild-type is indicated as: *, *p* < 0.05; **, *p* < 0.01; ***, *p* < 0.001.


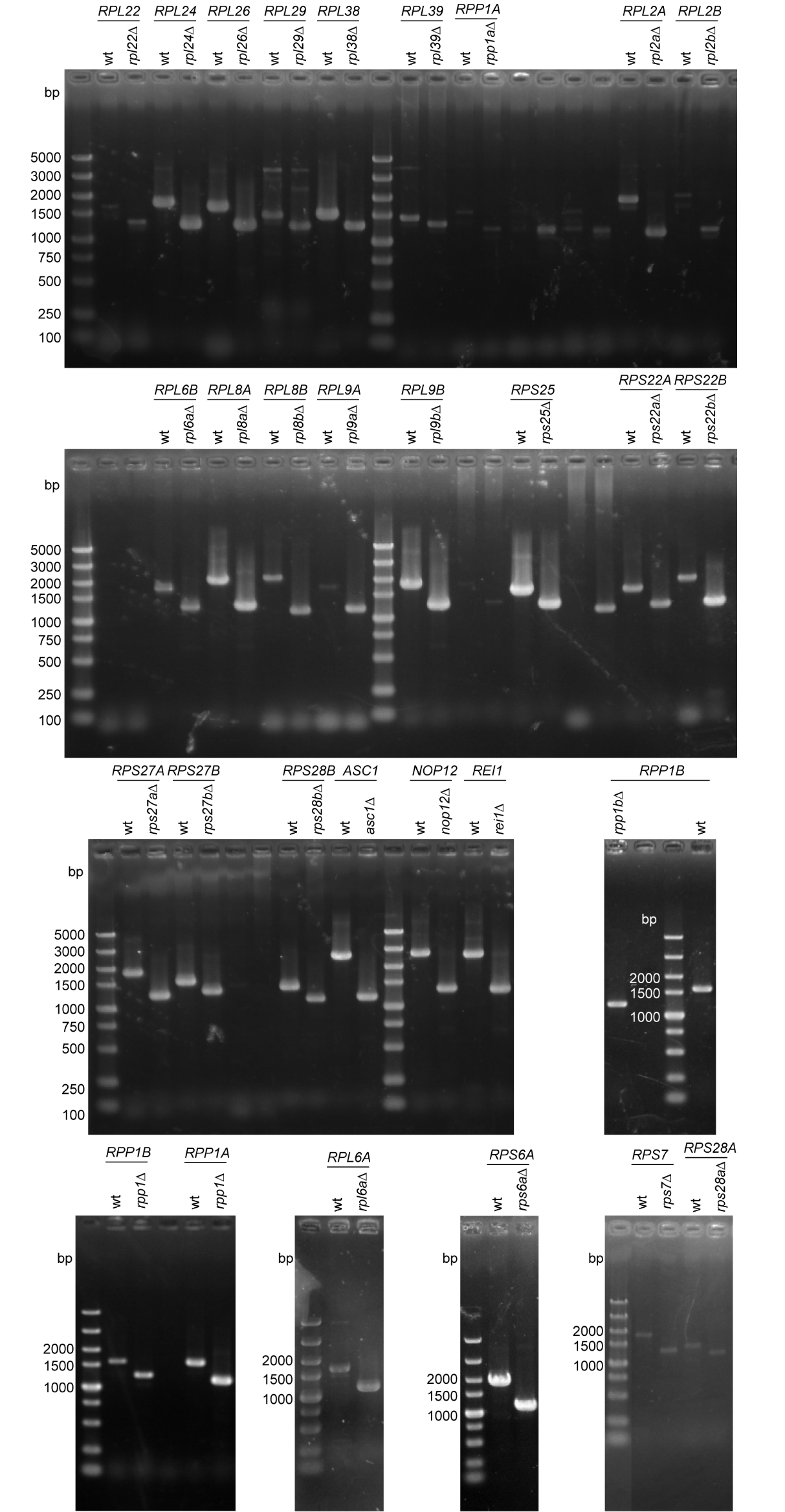


**Figure S1.** Confirmation of the deletants by PCR. After methanol induction, single colonies were isolated by streak plate method. Deletants were subject to PCR analysis using two outer primers (e.g. REI1-KO-S and REI1-KO-A), both of which were located outside of the homologous region. The PCR product was then analyzed by 1.5% agarose gel electrophoresis. An expected deleted fragment could be amplified from deletants using the wide type strains as control. The gene size of RPGs and 60 processing factors is as follows: *rpl22* (390bp), *rpl24* (477bp), *rpl26* (384bp), *rpl29* (180bp), *rpl38* (237bp), *rpl39* (156bp), *rpp1a* (321bp), *rpp1b* (324bp), *rpp1* (321/324bp), *rpl2a* (765bp), *rpl2b* (825bp), *rpl6a* (543bp), *rpl6b* (504bp), *rpl8a* (753bp), *rpl8b* (872bp), *rpl9a* (576bp), *rpl9b* (576bp), *rps7* (567bp), *rps25* (327bp), *rps6a* (717bp), *rps22a* (393bp), *rps22b* (686bp), *rps27a* (579bp), *rps27b* (249bp), *rps28a* (204bp), *rps28b* (204bp), *asc1* (1286bp), *nop12* (1338bp), *rei1* (1224bp).


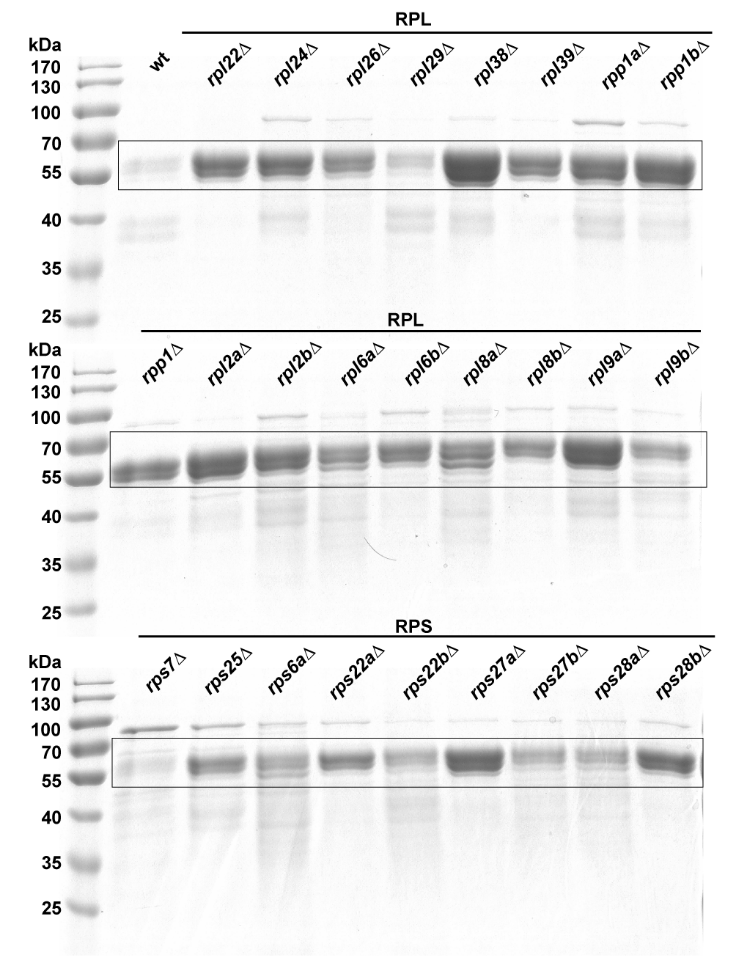


**Figure S2.** SDS-PAGE analysis of phytase expression with the collected culture supernatant in RPG deletion strains relative to wild-type at 120h induction time. The molecular weight (MW) of glycosylated Phy is approximately 55 kDa.


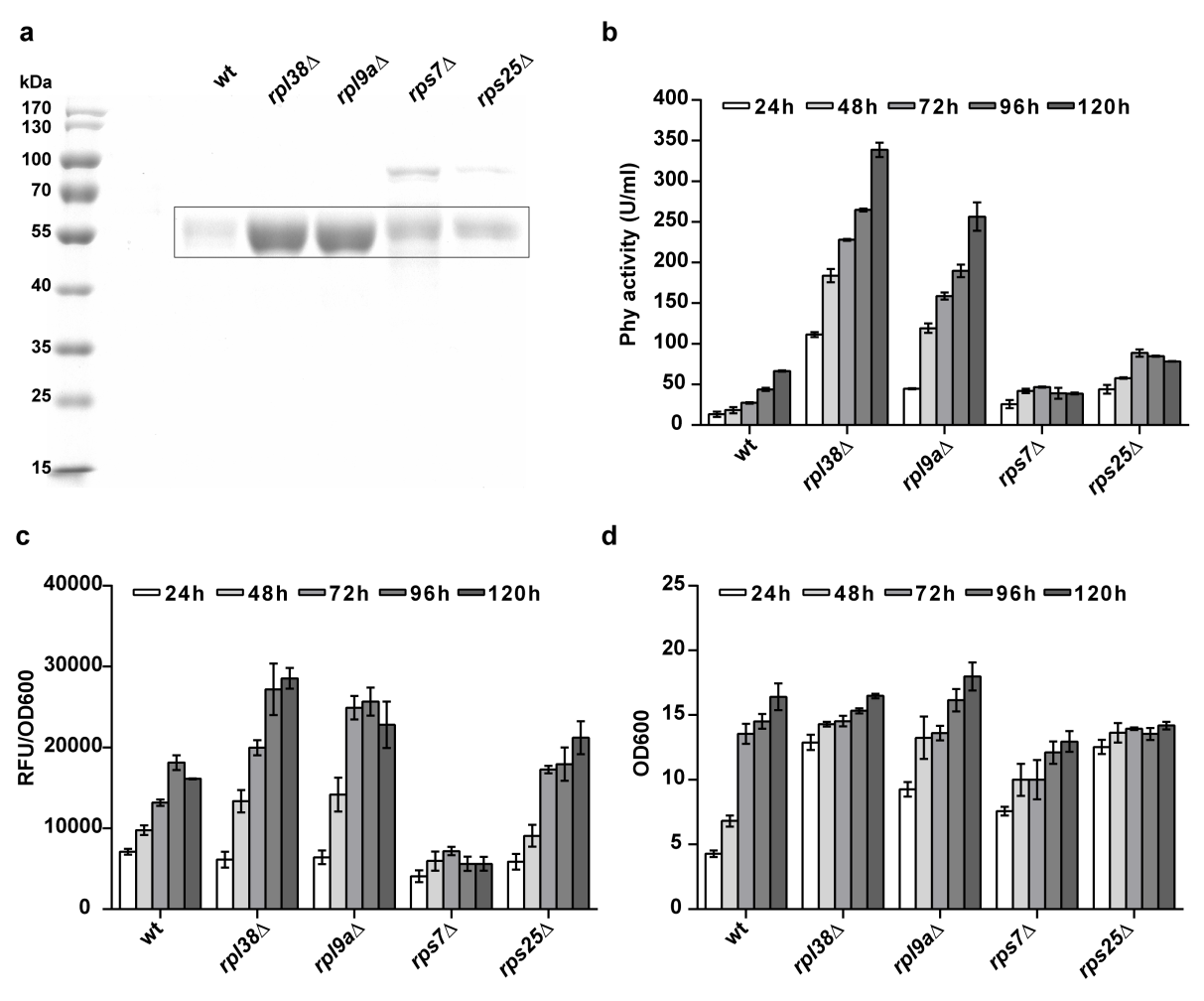


**Figure S3.** Expression analysis of two heterologous proteins in four RP deletion strains and wild-type. **a** SDS-PAGE analysis of Phy expression with the collected culture supernatant in *rpl38*∆, *rpl9a*∆, *rps7*∆, *rps25*∆ strains relative to wild-type at 120h induction time. **b** Phy expression profiles (activity per ml culture) of *rpl38*∆, *rpl9a*∆, *rps7*∆, *rps25*∆ strains relative to the wild-type strain. **c-d** eGFP expression profiles and growth curve of *rpl38*∆, *rpl9a*∆, *rps7*∆, *rps25*∆ strains in relation to wild-type strain.


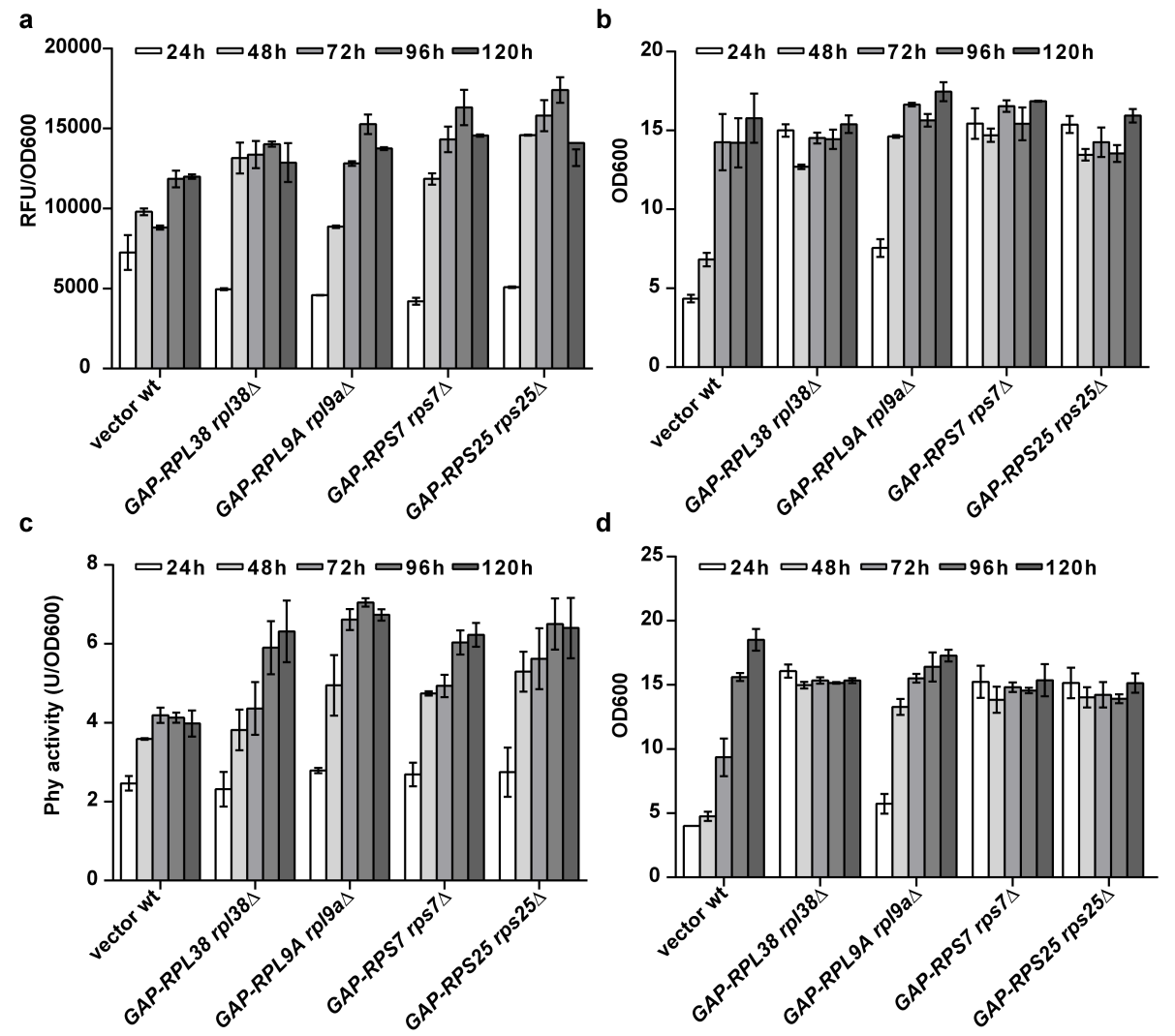


**Figure S4.** RP complementation assay with eGFP and Phy expression profiles in RP deletion strains relative to wild-type. **a-b** eGFP expression profiles and growth curve. **c-d** Phy expression profiles and growth curve. The *RPL38*, *RPL9A*, *RPS7*, *RPS25* gene were amplified by PCR using the primers shown as (Additional file 3: Table S5) from *P. pastoris* GS115 genome. Seamless Assembly Cloning Kit (C5891-25, CloneSmarter, USA) was used to clone these genes into the pGAPZA plasmid. The GAP-RP cassettes were amplified by the specific primers to assemble into the pPICZαA-eGFP and pPICZαA-Phy plasmid, respectively. Then RP deletion strains were transformed with the *Mss*I-linearized expression cassettes to yield RP-complemented strains.


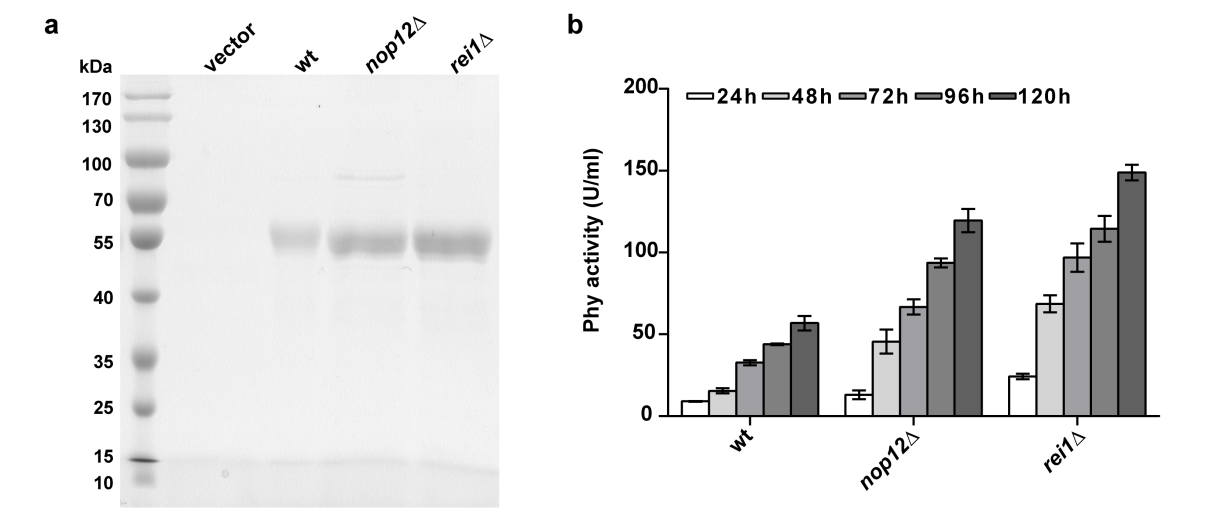


**Figure S5.** Phy expression profiles of the 60S processing factor deletion strains relative to the wild-type strain. **a** SDS-PAGE analysis of the collected culture supernatant at 120h induction time. **b** Activity per ml culture.


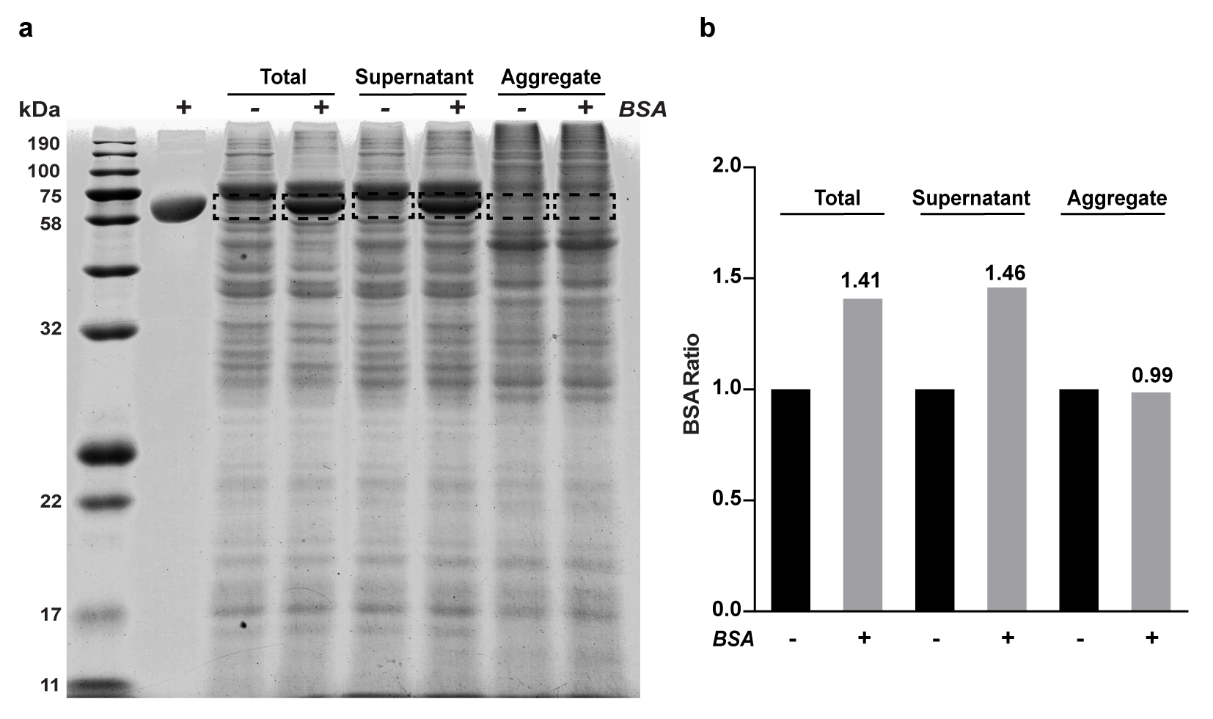


**Figure S6.** Validation of the method used in isolation of protein aggregates. **a** BSA removal assay. Aggregated protein extraction was performed with total protein and total protein mixed with 10% BSA, separately. SDS-PAGE assay was carried out to visualize the protein band. The first lane was loaded with pure BSA as positive control and the other lane with the dashed boxes indicated the bands of approximate MW of BSA. **b** Quantitative analysis of BSA removal efficiency. The BSA optical density ratios were revealed through normalizing the optical density values of the bands with known BSA additions to those without BSA additions.


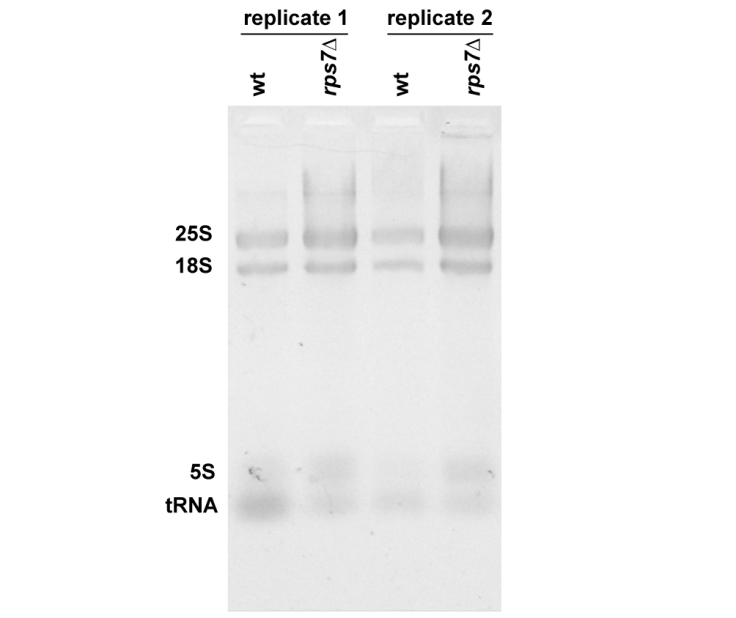


**Figure S7.** Semi-quantitative analysis of ribosomal RNA in *rps7*∆ strain and wild-type. After methanol induction for 12h, total RNA was extracted with the same OD600 of *rps7*∆ strain and wt. The concentrations of total RNA of the replicate 1: 749 ng/µl (wt), 1049 ng/µl (*rps7*∆), and the replicate 2: 738 ng/µl (wt), 1209 ng/µl (*rps7*∆) were measured using a Nano-drop spectrophotometer. Finally, the same volume of the sample was analyzed by 2.5% agarose gel electrophoresis.
