# Supplementary material for: Enhancing co-translational folding of heterologous protein by deleting non-essential ribosomal proteins in Pichia pastoris
Source: Biotechnol Biofuels. 2019 Feb 21;12:38. doi: 10.1186/s13068-019-1377-z (PMC6383220; doi:10.1186/s13068-019-1377-z)
Supplement: Supplementary file 3 — Additional file 3: Table S5. Primers used in this study. [file 13068_2019_1377_MOESM3_ESM.docx]

**Table S5. Primer used in this study.**

| Oligo # | Oligo name | Sequence 5’-3’ |
| --- | --- | --- |
| 1 | Cre-S | CCTTGGAATTCATGTCCAATTTACTGACC |
| 2 | Cre-A | CTAGGCCGCGGCTAATCGCCATCTTCCAG |
| 3 | lox71 | TACCGTTCGTATAGCATACATTATACGAAGTTATAGATCTAACATCCAAAGACG |
| 4 | lox66 | TACCGTTCGTATAATGTATGCTATACGAAGTTATGTCTCCAGCTTGCAAATTAA |
| 5 | Cre-Pc | CTTGCGAACCTCATCACTCGT |
| 6 | RPL12U-S | GTGGTCTTTCTTGTACAGCTTTGGAGTAT |
| 7 | RPL12U-A | AGATCTATAACTTCGTATAATGTATGCTATACGAACGGTACTTGTTTCTGTGAGGAGT |
| 8 | RPL12D-S | GGAGACATAACTTCGTATAGCATACATTATACGAACGGTAGATGGTTTGACTTCCAGC |
| 9 | RPL12D-A | GGTGCTTTAGTTAGAAGCCTGGTTAACCT |
| 10 | RPL12-KO-S | CCATCGGTTTTTCCGCCT |
| 11 | RPL12-KO-A | GCGCACGACTTTTGGGTGAT |
| 12 | RPL22U-S | TCGATAGTGTCCCTAGGTGGATCAACTAC |
| 13 | RPL22U-A | AGATCTATAACTTCGTATAATGTATGCTATACGAACGGTACCAGAGAAAGAGAAAGGC |
| 14 | RPL22D-S | GGAGACATAACTTCGTATAGCATACATTATACGAACGGTAGTCTATGGGTGTAATCAG |
| 15 | RPL22D-A | GTATTTATAAAGATCAGATATCCCTCATGAGT |
| 16 | RPL22-KO-S | GAAGTTGGGCCTGTAAACTCAGT |
| 17 | RPL22-KO-A | GTGATCCAAAATGCATAAGACTGAT |
| 18 | RPL24U-S | AGGGTACCAACTCTGAAGAGCTTACCACA |
| 19 | RPL24U-A | AGATCTATAACTTCGTATAATGTATGCTATACGAACGGTATGTTCCAGGTATAGCGAC |
| 20 | RPL24D-S | GGAGACATAACTTCGTATAGCATACATTATACGAACGGTAACGTCCGAAACCTATTGT |
| 21 | RPL24D-A | CTCCTACCAGAGGTAGATGCCATAGTTGT |
| 22 | RPL24-KO-S | TTTAGTTTCCAACAACGGAAAG |
| 23 | RPL24-KO-A | ATCTCCCTGCCCATCTCAATAT |
| 24 | RPL26U-S | AATGAGATATAAATCAGCCTTCAACTCGC |
| 25 | RPL26U-A | AGATCTATAACTTCGTATAATGTATGCTATACGAACGGTATTGTTTGAGTAAGGAGCG |
| 26 | RPL26D-S | GGAGACATAACTTCGTATAGCATACATTATACGAACGGTAACCGAAGTATCACCGAGT |
| 27 | RPL26D-A | GTCCAGTTTCTCCCATCTTGTTTGTTTCC |
| 28 | RPL26-KO-S | CGTACAAAGAACTTGCTCTCCA |
| 29 | RPL26-KO-A | AATCGACACTGTCGTGAATTCC |
| 30 | RPL29U-S | CTTTGATTCATAGAAGCACAGCTACCGTT |
| 31 | RPL29U-A | AGATCTATAACTTCGTATAATGTATGCTATACGAACGGTAACTTAGTTGGGCTTTGCC |
| 32 | RPL29D-S | GGAGACATAACTTCGTATAGCATACATTATACGAACGGTATCACTGCGAGTAAAGGAT |
| 33 | RPL29D-A | TTACTAATGGCAATGGAGTATCTCTCATTGG |
| 34 | RPL29-KO-S | GGGGGTTCCATATGCGATTG |
| 35 | RPL29-KO-A | CCCATGCAAAGTGTCCAGCA |
| 36 | RPL31U-S | TGGATGAATTGTCCACATCC |
| 37 | RPL31U-A | AGATCTATAACTTCGTATAATGTATGCTATACGAACGGTA AGGTAACGGAAGGTTTAG |
| 38 | RPL31D-S | GGAGACATAACTTCGTATAGCATACATTATACGAACGGTATGAATTCTTGGAAGGGCG |
| 39 | RPL31D-A | GGTAAGAAAACTTCCTCACC |
| 40 | RPL31-KO-S | GCTGATTGTACACTACGAAACC |
| 41 | RPL31-KO-A | CTGAAGGGATTGTTGGAACC |
| 42 | RPL38U-S | TGTCACCATATCCGCAGTAA |
| 43 | RPL38U-A | AGATCTATAACTTCGTATAATGTATGCTATACGAACGGTATTGGTGTGTGTGTTGGTG |
| 44 | RPL38D-S | GGAGACATAACTTCGTATAGCATACATTATACGAACGGTACGGACATAAACTGAAAGG |
| 45 | RPL38D-A | TCTATTACGATAACACCTGG |
| 46 | RPL38-KO-S | CATCCCTGTTTGAAATCATC |
| 47 | RPL38-KO-A | GTCATCCAAACCAAGGTCTA |
| 48 | RPL39U-S | TCGTGGTCCATAATACCGGC |
| 49 | RPL39U-A | AGATCTATAACTTCGTATAATGTATGCTATACGAACGGTAATGTGGTCTGGTAAGTTG |
| 50 | RPL39D-S | GGAGACATAACTTCGTATAGCATACATTATACGAACGGTAGTTAGCCAGCTTCTATTC |
| 51 | RPL39D-A | TTGCCAACATGAAACCGC |
| 52 | RPL39-KO-S | CACATCTAGTAGACGAAACC |
| 53 | RPL39-KO-A | GAACATGTCAGTTGACAG |
| 54 | RPP1AU-S | CCATAGAAGAACTGGATG |
| 55 | RPP1AU-A | AGATCTATAACTTCGTATAATGTATGCTATACGAACGGTATTGCGGACTAATCCCTTG |
| 56 | RPP1AD-S | GGAGACATAACTTCGTATAGCATACATTATACGAACGGTAATGTACCTTGCCTATGCC |
| 57 | RPP1AD-A | TGAGTTGAACGTCCAAAG |
| 58 | RPP1A-KO-S | ATTAATGCCATCCCACCG |
| 59 | RPP1A-KO-A | ACCGTTGGTATTGCTGTC |
| 60 | RPP1BU-S | CTGAAAGAGTATCGCTCT |
| 61 | RPP1BU-A | AGATCTATAACTTCGTATAATGTATGCTATACGAACGGTACTAGTTATGGTCTAGCGA |
| 62 | RPP1BD-S | GGAGACATAACTTCGTATAGCATACATTATACGAACGGTAGTAAGCTATGAACCCAAG |
| 63 | RPP1BD-A | AGGAACGTTTGCAAGAGT |
| 64 | RPP1B-KO-S | GCTATGATTGCTGAACTC |
| 65 | RPP1B-KO-A | TCGTCAAGCCACCAACCA |
| 66 | RPP2U-S | TCAGAGCGGCAAATCTGG |
| 67 | RPP2U-A | AGATCTATAACTTCGTATAATGTATGCTATACGAACGGTAACACCACACTCGTCTCAG |
| 68 | RPP2D-S | GGAGACATAACTTCGTATAGCATACATTATACGAACGGTA GGCGAATAGAGATGATTG |
| 69 | RPP2D-A | AGCTCCAGTGGTTGGATC |
| 70 | RPP2-KO-S | GATAGTGGGAGTACTTTG |
| 71 | RPP2-KO-A | GGGTTAGCAGGAGAAGAT |
| 72 | RPP0U-S | ATGTTGACCTCAGTCGTC |
| 73 | RPP0U-A | AGATCTATAACTTCGTATAATGTATGCTATACGAACGGTACTATACTGAGGATAGGCT |
| 74 | RPP0D-S | GGAGACATAACTTCGTATAGCATACATTATACGAACGGTACACACCAAAATTGGGTTC |
| 75 | RPP0D-A | TTTGCTCAAGTTGACCTC |
| 76 | RPP0-KO-S | CAACGAAGTCAGCACCGT |
| 77 | RPP0-KO-A | ATGATAGCCTTTTACCCG |
| 78 | RPL2AU-S | CCAGAGACTTTCAGTCAG |
| 79 | RPL2AU-A | AGATCTATAACTTCGTATAATGTATGCTATACGAACGGTAGTTTCTTGCTGTAAGAGG |
| 80 | RPL2AD-S | GGAGACATAACTTCGTATAGCATACATTATACGAACGGTATTAGGTGAGTAGTAGGAG |
| 81 | RPL2AD-A | AAAGCAGAGGAAGATCAG |
| 82 | RPL2A-KO-S | AGGGTGTTAAGAAGAACG |
| 83 | RPL2A-KO-A | TCTTCCCCAGAGAGTAGC |
| 84 | RPL2BU-S | TACCCCGTCATGTTGTTC |
| 85 | RPL2BU-A | AGATCTATAACTTCGTATAATGTATGCTATACGAACGGTATTCACCTAAGATGGAAGG |
| 86 | RPL2BD-S | GGAGACATAACTTCGTATAGCATACATTATACGAACGGTATGTAGCTTTTCGTAGAGC |
| 87 | RPL2BD-A | GCGTAGAGCTTTCTGTGA |
| 88 | RPL2B-KO-S | GTTTCGATCCCCAGAAGG |
| 89 | RPL2B-KO-A | CGGTTGTGTTTCATCCTT |
| 90 | RPL6AU-S | CTGCCATGTTACTCTCGT |
| 91 | RPL6AU-A | AGATCTATAACTTCGTATAATGTATGCTATACGAACGGTAGATCCAAGGTCAATGGTC |
| 92 | RPL6AD-S | GGAGACATAACTTCGTATAGCATACATTATACGAACGGTAAGGGTAGACAATGCTGTG |
| 93 | RPL6AD-A | ACTGTCGGATTCATCTCC |
| 94 | RPL6A-KO-S | CATCAGTTAATGACCGTC |
| 95 | RPL6A-KO-A | TCCTCCTCTGCGTCCTCT |
| 96 | RPL6BU-S | CCTTCTTCTAAAGTACCG |
| 97 | RPL6BU-A | AGATCTATAACTTCGTATAATGTATGCTATACGAACGGTAAAACCTTGGTTGGAAACG |
| 98 | RPL6BD-S | GGAGACATAACTTCGTATAGCATACATTATACGAACGGTAACCTGTGACTCTATCCCC |
| 99 | RPL6BD-A | GAACAAGACTTTGCCCTC |
| 100 | RPL6B-KO-S | TTGCCATAGAAGCTTGGA |
| 101 | RPL6B-KO-A | TCTCAAAAACGACAGGAG |
| 102 | RPL8AU-S | GCACCAATTAGGTTGGCA |
| 103 | RPL8AU-A | AGATCTATAACTTCGTATAATGTATGCTATACGAACGGTATAACAGCTAGAACTCCTC |
| 104 | RPL8AD-S | GGAGACATAACTTCGTATAGCATACATTATACGAACGGTAGAGCATTAGAAAGAGGAC |
| 105 | RPL8AD-A | CTAAGCAGTCTTAGCAGC |
| 106 | RPL8A-KO-S | GTAACAAGTTGGCCCAGA |
| 107 | RPL8A-KO-A | GATCCTTTACTCGCAGTG |
| 108 | RPL8BU-S | CTCTTTTCGTCTGATTGG |
| 109 | RPL8BU-A | AGATCTATAACTTCGTATAATGTATGCTATACGAACGGTAAGCTAGTAGGTGAAACCG |
| 110 | RPL8BD-S | GGAGACATAACTTCGTATAGCATACATTATACGAACGGTAGAGCTGGTTCTGGTTTCC |
| 111 | RPL8BD-A | CACCTGACACTGCTATAG |
| 112 | RPL8B-KO-S | GCTTCCTGTTCACCTTCA |
| 113 | RPL8B-KO-A | AAATTCGCGAACGGTGAA |
| 114 | RPL9AU-S | GTTCAGTTCAACTGAGAG |
| 115 | RPL9AU-A | AGATCTATAACTTCGTATAATGTATGCTATACGAACGGTAGGGGATATATAGGAAGCA |
| 116 | RPL9AD-S | GGAGACATAACTTCGTATAGCATACATTATACGAACGGTAGTTAGGCTTTCCACACTG |
| 117 | RPL9AD-A | ACCGTGGAACAAGAAAAG |
| 118 | RPL9A-KO-S | GTTTGAAGCTTGTTCGGG |
| 119 | RPL9A-KO-A | ACTAGCAGACGATCAATC |
| 120 | RPL9BU-S | CGTCTAGGGAATGTCTCT |
| 121 | RPL9BU-A | AGATCTATAACTTCGTATAATGTATGCTATACGAACGGTATTTCACACTCTAACGGGC |
| 122 | RPL9BD-S | GGAGACATAACTTCGTATAGCATACATTATACGAACGGTATGACATGGAGTTGTAGAG |
| 123 | RPL9BD-A | TGACCAGGTGAGGTTTAC |
| 124 | RPL9B-KO-S | ATGTGGTCTTTACTTCCC |
| 125 | RPL9B-KO-A | TATTGGTACCAAAGCCAG |
| 126 | RPS7U-S | GGTGTCAATTTCATCAGC |
| 127 | RPS7U-A | AGATCTATAACTTCGTATAATGTATGCTATACGAACGGTAGTGATACTGCTGTTACCG |
| 128 | RPS7D-S | GGAGACATAACTTCGTATAGCATACATTATACGAACGGTATTCTACGAAGCGTCGTTG |
| 129 | RPS7D-A | GTTTTTCACCTCTGGAGG |
| 130 | RPS7-KO-S | CTTGATAAAGCGCTGCCC |
| 131 | RPS7-KO-A | CTGCATCTCCCCAGTTTC |
| 132 | RPS12U-S | CACTGAGGCCACACTCTT |
| 133 | RPS12U-A | AGATCTATAACTTCGTATAATGTATGCTATACGAACGGTACGAGTAGTAGACTTCTTC |
| 134 | RPS12D-S | GGAGACATAACTTCGTATAGCATACATTATACGAACGGTAGTCAATTGTAGATGGTGC |
| 135 | RPS12D-A | ACCTGCAAGATCTTTCTG |
| 136 | RPS12-KO-S | CCAATATGCCTTCTTCCG |
| 137 | RPS12-KO-A | AACATCAATACCCAAGCC |
| 138 | RPS25U-S | AGGAGGTTTATGTTCGCG |
| 139 | RPS25U-A | AGATCTATAACTTCGTATAATGTATGCTATACGAACGGTAGACTGAAATAATCCTCGG |
| 140 | RPS25D-S | GGAGACATAACTTCGTATAGCATACATTATACGAACGGTAGAATAGTCTTCCTTGTCG |
| 141 | RPS25D-A | AACTTTCAGTCGGAACAC |
| 142 | RPS25-KO-S | AACCAGCTCTCCTCTTTC |
| 143 | RPS25-KO-A | TAGTCTCAGAAGCAGAAC |
| 144 | RPS6AU-S | TCAACATGAGGAGATTGG |
| 145 | RPS6AU-A | AGATCTATAACTTCGTATAATGTATGCTATACGAACGGTAATTTGACAAGCGCGAAGG |
| 146 | RPS6AD-S | GGAGACATAACTTCGTATAGCATACATTATACGAACGGTATAGATCTTCTGAGCAAGC |
| 147 | RPS6AD-A | TATGGCCTTTCAAACCCG |
| 148 | RPS6A-KO-S | CATTTGTAGAGGGAGTTG |
| 149 | RPS6A-KO-A | CACCAAACGATATGTTGG |
| 150 | RPS6BU-S | CTACTGCTCAATCTTTCC |
| 151 | RPS6BU-A | AGATCTATAACTTCGTATAATGTATGCTATACGAACGGTAGGTGCAAGATAACGCTTA |
| 152 | RPS6BD-S | GGAGACATAACTTCGTATAGCATACATTATACGAACGGTACTGTGGTTTATGAGATCC |
| 153 | RPS6BD-A | TTGATCGAAGAGGCCCCA |
| 154 | RPS6B-KO-S | GAAACACTCCATTAGCCC |
| 155 | RPS6B-KO-A | TGAGGAAAAGAGTTCGGG |
| 156 | RPS22AU-S | CCGTGTTTGAAGAAGCAG |
| 157 | RPS22AU-A | AGATCTATAACTTCGTATAATGTATGCTATACGAACGGTAATTCGATAGGTGTGATGG |
| 158 | RPS22AD-S | GGAGACATAACTTCGTATAGCATACATTATACGAACGGTATGGATTGTAGTTACAGGC |
| 159 | RPS22AD-A | TTGAGCTGCCAACATGGC |
| 160 | RPS22A-KO-S | CTCTCATGGAAAAGCCTA |
| 161 | RPS22A-KO-A | CTTTGCACTTAGCAGCAA |
| 162 | RPS22BU-S | AGCTCTAAATTGGGGTAC |
| 163 | RPS22BU-A | AGATCTATAACTTCGTATAATGTATGCTATACGAACGGTATTCCTAAGGCTACATGAG |
| 164 | RPS22BD-S | GGAGACATAACTTCGTATAGCATACATTATACGAACGGTATGCGTAGATGTTGGAACC |
| 165 | RPS22BD-A | CTAACAAAGAGGAAAGGG |
| 166 | RPS22B-KO-S | GTCAATTGAGCAGAAAGC |
| 167 | RPS22B-KO-A | CCCTAAGCTTGAAACTAG |
| 168 | RPS27AU-S | CAGAGATGGATAGCAATC |
| 169 | RPS27AU-A | AGATCTATAACTTCGTATAATGTATGCTATACGAACGGTAAAATTGTCACAAGGTGGG |
| 170 | RPS27AD-S | GGAGACATAACTTCGTATAGCATACATTATACGAACGGTAAGATTGCTGATGTAAGCC |
| 171 | RPS27AD-A | CTTGAAACTTACGCACAG |
| 172 | RPS27A-KO-S | CGGGTCAACAATGGTGAG |
| 173 | RPS27A-KO-A | TCCTTGCTCATTCTTCAC |
| 174 | RPS27BU-S | TTCAGTTTGGATGGAAGG |
| 175 | RPS27BU-A | AGATCTATAACTTCGTATAATGTATGCTATACGAACGGTAGGTGTAGGATACTTGATG |
| 176 | RPS27BD-S | GGAGACATAACTTCGTATAGCATACATTATACGAACGGTAAGTGGAACGATCAAGAGG |
| 177 | RPS27BD-A | AGACAAGCAGGACACGAC |
| 178 | RPS27B-KO-S | TGTCATGACTCGAATGTC |
| 179 | RPS27B-KO-A | ACTGACGCGTGTAATATC |
| 180 | RPS28AU-S | AGAGAGGATGAAAGTGCA |
| 181 | RPS28AU-A | AGATCTATAACTTCGTATAATGTATGCTATACGAACGGTAGAAATTTCAAGAGGCACG |
| 182 | RPS28AD-S | GGAGACATAACTTCGTATAGCATACATTATACGAACGGTATGATTATGCAGAAGGGTC |
| 183 | RPS28AD-A | AGTACAGTTGGGATTCCC |
| 184 | RPS28A-KO-S | GATGCTGAGGAGACTGAG |
| 185 | RPS28A-KO-A | CTATCAATCTAGCTCCAC |
| 186 | RPS28BU-S | CCCAAATCACATTTACCC |
| 187 | RPS28BU-A | AGATCTATAACTTCGTATAATGTATGCTATACGAACGGTAGTTTCTGTAGGTTCTGGG |
| 188 | RPS28BD-S | GGAGACATAACTTCGTATAGCATACATTATACGAACGGTATTTGATCATCGTGAGACG |
| 189 | RPS28BD-A | GCAGAAACATCCCCACAA |
| 190 | RPS28B-KO-S | TTCCTGAACCTGGTCAAT |
| 191 | RPS28B-KO-A | TCAGTTCTAGCCTTTCAC |
| 192 | ASC1U-S | GTTCCCATAAAAGTCGTG |
| 193 | ASC1U-A | AGATCTATAACTTCGTATAATGTATGCTATACGAACGGTACAACGATCGAGCAGAACG |
| 194 | ASC1D-S | GGAGACATAACTTCGTATAGCATACATTATACGAACGGTAGATTCTTGTAGCTGTTCG |
| 195 | ASC1D-A | GTACTTAATGGCGATGGG |
| 196 | ASC1-KO-S | CCCCGCTTATCTGGTTGT |
| 197 | ASC1-KO-A | CCTTTTGCTGCGTATGGG |
| 198 | NOP12U-S | CTCTGCCCTTTCTTGCTC |
| 199 | NOP12U-A | AGATCTATAACTTCGTATAATGTATGCTATACGAACGGTAGGCGTCAAAGCTGGTTAT |
| 200 | NOP12D-S | GGAGACATAACTTCGTATAGCATACATTATACGAACGGTAGAACCAACTAACGGCTGC |
| 201 | NOP12D-A | GAACACATTGGTGCTTGC |
| 202 | NOP12-KO-S | TCGTCAACTCGGGGTCTT |
| 203 | NOP12-KO-A | AATCCAACGAAAACTTGG |
| 204 | REI1U-S | GGACGCATTAGTGCCCTG |
| 205 | REI1U-A | AGATCTATAACTTCGTATAATGTATGCTATACGAACGGTAAGTGGAGTATAACGAGGG |
| 206 | REI1D-S | GGAGACATAACTTCGTATAGCATACATTATACGAACGGTATCAATGCGATGTTGGATG |
| 207 | REI1D-A | GACTCTACAAAGTCTGGG |
| 208 | REI1-KO-S | TGACGAAAACACAGATGG |
| 209 | REI1-KO-A | ACCACTTTCCCTCGCATA |
| 210 | pGAPZA-A | CACGTGAATTCCTCGTTTCG |
| 211 | pGAPZA-S | TTTAGCCTTAGACATGACTG |
| 212 | pGAP-Rpl38-S | CGAAACGAGGAATTCACGTGATGCCTAGACAAATCACTGA |
| 213 | pGAP-Rpl38-A | CAGTCATGTCTAAGGCTAAACTACAACTTGGTAACCTTCA |
| 214 | pGAP-Rpl9a-S | CGAAACGAGGAATTCACGTGATGAAGTACGTTTTATCCGAGC |
| 215 | pGAP-Rpl9a-A | CAGTCATGTCTAAGGCTAAATTATTCTTCTTGAACGATAGTTCCCTTC |
| 216 | pGAP-Rps7-S | CGAAACGAGGAATTCACGTGATGTCATCAGCCAAAATCCT |
| 217 | pGAP-Rps7-A | CAGTCATGTCTAAGGCTAAATTATAAAGTTTCACTTGGGATCTCGAAG |
| 218 | pGAP-Rps25-S | CGAAACGAGGAATTCACGTGATGCCACCAAAGATTCAACAATCC |
| 219 | pGAP-Rps25-A | CAGTCATGTCTAAGGCTAAATTACTCGGAAGCAGTAGCTCTAGTGTATATCT |
| 220 | pGAP-RP-S | CAGTGAGGTTATTTCTGAGCAGATCTTTTTTGTAGAAATGTCTTGGTG |
| 221 | pGAP-RP-A | TGGAGGACCATGTAAGTGGTGGATCCGCACAAACGAAGGT |
| 222 | pPICZαA-S | ACCACTTACATGGTCCTCCAGATCCCCCACACACCATA |
| 223 | pPICZαA-A | GCTCAGAAATAACCTCACTGTCTCACTTAATCTTCTGTACTCTG |

Naming of the primers is as follows: REI1U-S and REI1U-A, primers used in the amplification of the upstream homologous regions; REI1D-S and REI1D-A, primers used in the amplification of the downstream homologous regions; REI1-KO-S and REI1-KO-A, primers used in the analysis of the *rei1* deletion strain by PCR and sequencing.
